# Efficacy and Safety of Lipid-Lowering Drugs of Different Intensity on Clinical Outcomes: A Systematic Review and Network Meta-Analysis

**DOI:** 10.3389/fphar.2021.713007

**Published:** 2021-10-21

**Authors:** Wenrui Ma, Qinyuan Pan, Defeng Pan, Tongda Xu, Hong Zhu, Dongye Li

**Affiliations:** ^1^ Institute of Cardiovascular Disease Research, Xuzhou Medical University, Xuzhou, China; ^2^ Xuzhou Medical University Affiliated Hospital, Xuzhou, China

**Keywords:** lipid-lowering drugs, all-cause mortality, cardiovascular diseases, PCSK9 inhibitors, network meta-analysis

## Abstract

There have been many meta-analyses for statins, ezetimibe and proprotein convertase subtilisin/kexin type 9 inhibitors (PCSK9i) to evaluate clinical outcomes, but the efficacy and safety of different intensity of these three drugs on clinical outcomes was absent. PCSK9i, ezetimibe, and statins were divided into seven interventions as follows: including PCSK9i + high-intensity statins (P9i+HT), PCSK9i + moderate-intensity statins (P9i+MT), ezetimibe + high-intensity statins (Eze+HT), ezetimibe + moderate-intensity statins (Eze+MT), high-intensity statins (HT), moderate-intensity statins (MT), and low-intensity statins (LT). The risk ratios (RR) and 95% confidence intervals (CI) were calculated to evaluate the clinical outcomes in all randomized controlled trials included. In traditional meta-analysis, the more intensive treatment had a lower risk of all-cause mortality (RR 0.91, 95% CI 0.88–0.95), cardiovascular mortality (RR 0.89, 95% CI 0.86–0.92), myocardial infarction (RR 0.79, 95% CI 0.77–0.81), coronary revascularization (RR 0.80, 95% CI 0.76-0.84), and cerebrovascular events (RR 0.84, 95% CI 0.80–0.88) compared with the less intensive treatment. However, the more intensive treatment had a higher risk of new-onset diabetes (RR 1.08, 95% CI 1.04-1.12). The network meta-analysis demonstrated that P9i+HT, P9i+MT, HT, and MT were significantly associated with a risk reduction in coronary revascularization and cerebrovascular events compared with PLBO. LT could effectively reduce the risk of cardiovascular mortality (RR 0.71, 95% CI 0.54–0.92), MI (RR 0.67, 95% CI 0.54-0.82), and coronary revascularization (RR 0.77, 95% CI 0.65–0.91) compared with PLBO. P9i+HT was superior to HT in reducing the risk of MI (RR 0.78, 95% CI 0.68–0.90), coronary revascularization (RR 0.84, 95% CI 0.73–0.96), and cerebrovascular events (RR 0.78, 95% CI 0.64–0.95). However, compared with PLBO, P9i+HT, HT, and MT could increase the risk of new-onset diabetes (RR 1.23, 95% CI 1.11–1.37; RR 1.23, 95% CI 1.14–1.33; RR 1.09, 95% CI 1.02–1.15, respectively). In conclusion, PCSK9i added to background statins may be recommended as preferred lipid-lowering therapy, and did not increase the additional risk of new-onset diabetes. The safety and efficacy of ezetimibe was not superior to that of statins. LT can be recommended as the initial therapy.

## Introduction

Lipid-lowering drugs, which are pivotal in the treatment of atherosclerotic cardiovascular diseases (ASCVD), can reduce major adverse cardiovascular events (MACE) to a certain extent (Bonovas et al., 2011). Currently, the clinically recommended lipid-lowering drugs are statins, ezetimibe, and proprotein convertase subtilisin/kexin type 9 inhibitors (PCSK9i) (Ruscica et al., 2021). As the commonly used lipid-lowering drugs since the mid-1980s, statins can decrease low-density lipoprotein cholesterol (LDL-C) levels (Reiner, 2013). However, LDL-C levels cannot be reduced to ESC/EAS guidelines in many patients by statins-alone (Mach et al., 2020). Therefore, ezetimibe, an inhibitor of cholesterol absorption, is added to further reduce LDL-C levels within the background of statin therapy (Cannon et al., 2015). In addition, there are many patients whose LDL-C levels cannot be reduced to target levels, even with the addition of ezetimibe, but the use of PCSK9i can further reduce LDL-C levels and the risk of clinical outcomes (AlTurki et al., 2019). Moreover, similar clinical benefits can be achieved for PCSK9i, alirocumab and evolocumab, which are currently used clinically (Guedeney et al., 2021). For patients with hypercholesterolemia, the use of the PCSK9i greatly alleviates the harm caused by high LDL-C levels (Navarese et al., 2018; Reiner, 2018).

Previous meta-analyses have reported the direct comparison of two of the three drugs but did not clarify the effects of different drug types and their intensity on the clinical outcomes. In general, clinicians establish the drug protocol based on the patient’s LDL-C level. Currently, statins can be divided into three levels according to the degree to which they lower LDL-C levels, namely, high intensity, moderate intensity, and low intensity (Stone et al., 2014). Moderate-intensity statins are the most commonly used. A previous network meta-analysis reported that high-intensity statins can significantly reduce LDL-C levels but did not clarify the effects of statins of different intensity on clinical outcomes (Khan et al., 2018). Moreover, Toth et al. analyzed the effects of PCSK9i and ezetimibe on LDL-C levels but did not compare the clinical outcomes (Toth et al., 2017). Therefore, this network meta-analysis aims to determine which lipid-lowering therapy can achieve the best effect for different clinical outcomes.

## Methods

This network meta-analysis followed the PRISMA Statement (Moher et al., 2009) and was registered with PROSPERO (CRD42021244226).

### Objectives, Data Sources, and Search Strategies

This network meta-analysis evaluated the effects of three types of commonly used lipid-lowering drugs on clinical outcomes. We searched embase, PubMed, and Cochrane databases up to March 2021 and for terms relevant to statins, ezetimibe, PCSK9 inhibitors, and randomized controlled trials. The search entries were adjusted to apply to each database, and all terms were imported into Endnote X9 for manual screening according to the inclusion criteria (Supplementary Table S1).

### Study Selection and Data Abstraction

Many studies related to lipid-lowering drugs are restricted by a small number of participants and short duration, which often brings uncertainty to the study results. We set the inclusion criteria to studies including >500 participants, lasting at least 48 weeks, and reporting one or more outcomes, such as all-cause mortality, cardiovascular mortality, myocardial infarction (MI), coronary revascularization, cerebrovascular events, cancer, and new-onset diabetes. The eligible trials included a comparison of at least one of the drugs, namely, statins, ezetimibe, and PCSK9i. Bococizumab, a PCSK9i, was excluded because of the existence of anti-drug antibodies and the attenuation of the lipid-lowering effect during treatment (Ridker et al., 2017). Two investigators independently assessed the terms to avoid bias in the data search and abstraction process. The opinion of a third investigator was sought in case of disagreement. We extracted the name and year of all studies, the total number of participants, the follow-up time, the intervention measures of the intervention and control group, the baseline and endpoint LDL-C levels, and the number of events with outcomes in the eligible studies. Studies with a follow-up time of at least 48 weeks were included because the clinical outcomes were divergent at approximately 1 year after drug use (Sever et al., 2003; Koren and Hunninghake, 2004).

### Interventions

The three drug categories in the selected studies were divided into seven interventions for comparison as follows: PCSK9i + high-intensity statins (P9i + HT), PCSK9i + moderate-intensity statins (P9i + MT), ezetimibe + high-intensity statins (Eze + HT), ezetimibe + moderate-intensity statins (Eze + MT), high-intensity statins (HT), moderate-intensity statins (MT), and low-intensity statins (LT).

### Data Analysis

STATA 13.0 software was used to conduct frequentist network meta-analysis. RR and 95% CI values were calculated to evaluate the clinical outcomes. We used the surface under the cumulative ranking curve (SUCRA) to rank which drugs could achieve the relatively better therapeutic effect in different clinical outcomes. The inconsistent model was used to test the data, and P-value > 0.05 indicated no inconsistency. The consistency of the direct and indirect comparison results was evaluated by the inconsistency factor (IF) and 95% CI. I^2^ values were determined to assess the statistical heterogeneity in the paired meta-analysis conducted by Review Manager 5.2 software. Heterogeneity existed if I^2^ ≥ 50%. The Cochrane Manual was used to evaluate the quality of each eligible trial, which was divided into high, unclear, and low risk of bias. Funnel plots were used to estimate the publication bias. In studies of PCSK9i, we used the intensity of the statins with the highest number of participants as the background statin intensity. When there were zero incidents in one arm of the study, we added 0.5 to each arm and performed the Haldane method (Sheehe, 1966).

## Results

**FIGURE 1:**
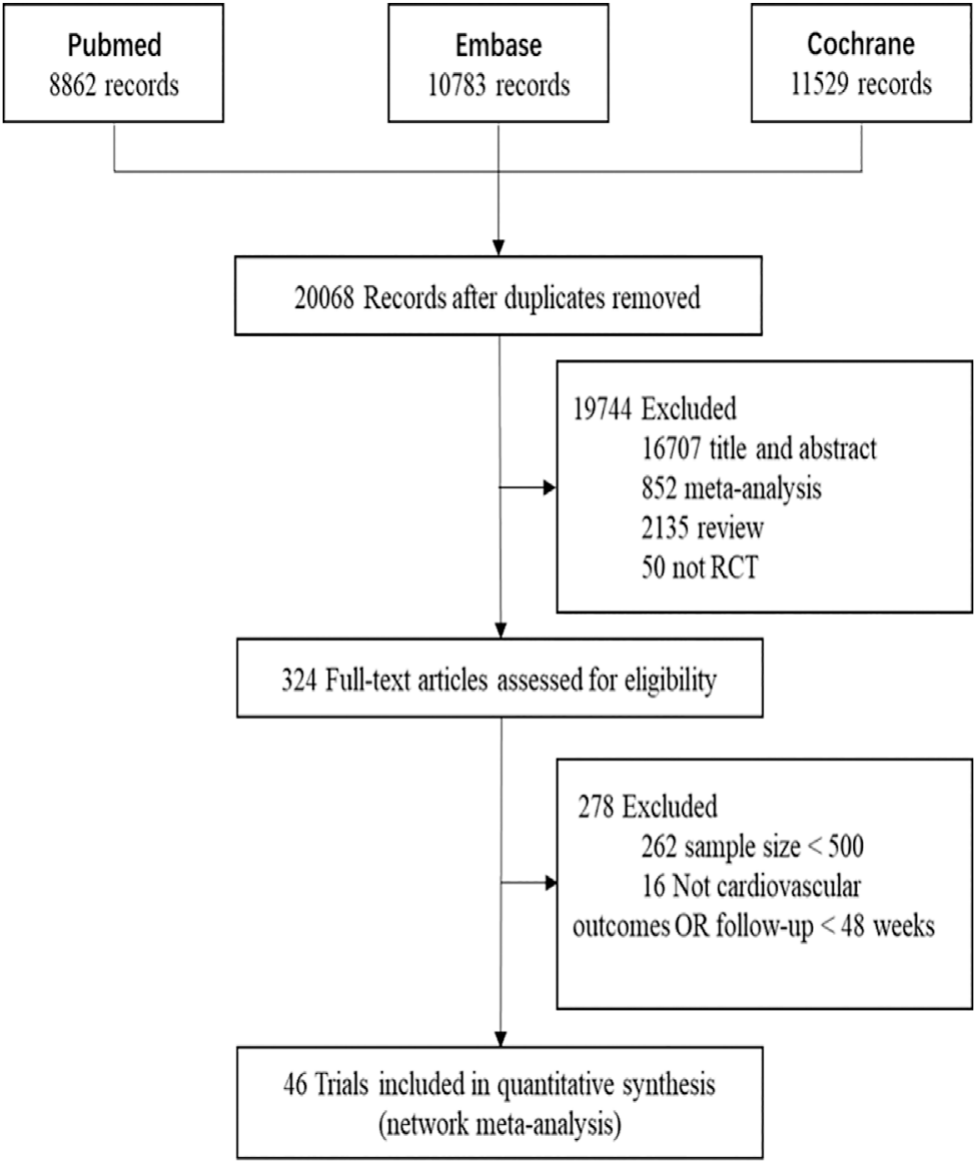
The process of search and selection of randomized controlled trials for network meta-analysis in the flow chart. RCT, randomized controlled trials.

In this network meta-analysis, 46 two-arm studies were included (Table 1). Figure 1 shows the search results of the three databases, the eliminative conditions, and the trials included for analysis. Among the eligible studies, the intervention group was comprised of five studies of P9i + HT, one study of P9i + MT, one study of Eze + HT, four studies of Eze + MT, 11 studies of HT, 18 studies of MT, and six studies of LT. Most of the selected studies were secondary prevention and mixed prevention in nature, and the mean follow-up time was 3.61 years.

**TABLE 1:** Characteristics of trials final included in the network meta-analysis.

Study	Total patients	Follow-up (years)	Intervention			Control		
			Treatment	Baseline LDL-C (mmol/L)	Achieved LDL-C (mmol/L)	Treatment	Baseline LDL-C (mmol/L)	Achieved LDL-C (mmol/L)
Wanner et al. (2005)	1,255	4	Atorvastatin 20 mg	3.2	1.9	Placebo	3.3	3.1
4S. Group (1994)	4,444	5.4	Simvastatin 20–40 mg	4.9	3.2	Placebo	4.9	4.8
de Lemos et al. (2004)	4,497	2	Simvastatin 80 mg	2.9	1.7	Simvastatin 20 mg	2.9	2.1
Downs et al. (1998)	6,605	5.2	Lovastatin 20–40 mg	3.9	3.0	Placebo	3.9	3.7
Holdaas et al. (2003)	2,102	5.1	Fluvastatin 40–80 mg	4.1	2.8	Placebo	4.1	3.8
T. A. O. a. C. f. t. A. C. R. Group (2002)	10,355	4.8	Pravastatin 40 mg	3.8	2.7	Usual care	3.8	3.6
Koren and Hunninghake (2004)	2,442	4.3	Atorvastatin 40–80 mg	3.8	2.5	Usual care	3.8	2.9
Sever et al. (2003)	10,305	3.3	Atorvastatin 10 mg	3.4	2.2	Placebo	3.4	3.2
Knopp et al. (2006)	2,410	4.25	Atorvastatin 10 mg	2.9	2	Placebo	3.0	2.9
Fellström et al. (2009)	2,773	3.2	Rosuvastatin 10 mg	2.6	1.5	Placebo	2.6	2.6
Colhoun et al. (2004)	2,841	4	Atorvastatin 10 mg	3.0	2.1	Placebo	3.1	2.6
Sacks et al. (1996)	4,159	5	Pravastatin 40 mg	3.6	2.5	Placebo	3.6	3.5
Kjekshus et al. (2007)	5,011	3	Rosuvastatin 10 mg	3.6	2.0	Placebo	3.6	3.6
Kastelein et al. (2008)	720	2	Ezetimibe 10 mg + simvastatin 80 mg	8.2	3.7	Simvastatin 80 mg	8.2	5.0
Liem et al. (2002)	540	1	Fluvastatin 80 mg	3.5	2.7	Placebo	3.6	3.9
Sabatine et al. (2017)	27,564	2.2	Evolocumab	2.4	0.8	high statins	2.4	2.4
Tavazzi et al. (2008)	4,574	3.9	Rosuvastatin 10 mg	3.9	3.3	Placebo	3.9	3.8
Investigators (2000)	4,271	2	Pravastatin 20 mg	3.9	3.3	Usual care	3.9	3.8
Nicholls et al. (2016)	968	1.5	Evolocumab	2.4	0.9	high statins	2.4	2.4
Athyros et al. (2002)	1,600	3	Atorvastatin 10–80 mg	4.7	2.5	Usual care	4.7	4.4
Hagiwara et al. (2017)	1721	3.9	Ezetimibe 10 mg + Pitavastatin 2 mg	3.5	1.7	Pitavastatin 2 mg	3.5	2.2
Yusuf et al. (2016)	12,705	5.6	Rosuvastatin 10 mg	3.3	2.4	Placebo	3.3	3.2
ALLHAT Collaborative Research Group. (2002)	20,536	5	Simvastatin 40 mg	3.4	2.4	Placebo	3.4	3.1
Pedersen et al. (2005)	8,888	4.8	Atorvastatin 80 mg	3.1	2.1	Simvastatin 20 mg	3.1	2.6
Cannon et al. (2015)	18,144	6	Ezetimibe 10 mg + Simvastatin 40 mg	2.4	1.3	Simvastatin 40 mg	2.4	1.8
Hosomi et al. (2015)	1,578	4.9	Pravastatin 10 mg	3.4	2.7	Placebo	3.4	3.2
Ridker et al. (2008)	17,802	4	Rosuvastatin 20 mg	2.8	1.5	Placebo	2.8	2.8
L. S. GROUP (1998)	9,014	6.1	Pravastatin 40 mg	3.9	2.9	Placebo	3.9	3.9
Serruys et al. (2002)	1,677	3.9	Fluvastatin 80 mg	3.4	2.5	Placebo	3.4	3.0
Nakamura et al. (2006)	7,832	5.3	Pravastatin 10–20 mg	4.1	3.3	Diet	4.1	3.7
Crouse et al. (2007)	984	2	Rosuvastatin 40 mg	4.0	2.0	Placebo	4.0	4.0
Cannon et al. (2015)	720	2	Alirocumab	2.8	1.3	Ezetimibe	2.7	2.2
Robinson et al. (2015)	2,341	1.5	Alirocumab	3.1	1.5	high statins	3.1	3.2
Schwartz et al. (2018)	18,924	2.8	Alirocumab	2.4	1.4	high statins	2.4	2.7
Sabatine et al. (2015)	4,465	0.9	Evolocumab	3.1	1.2	Moderate statins	3.1	3.1
Patients (1993)	1,062	1	Pravastatin 20 mg	4.7	3.5	Placebo	4.7	4.7
Shepherd et al. (2002)	5,804	3.2	Pravastatin 40 mg	3.8	2.5	Placebo	3.8	3.6
Cannon et al. (2004)	4,162	2	Atorvastatin 80 mg	2.7	1.6	Pravastatin 40 mg	2.7	2.5
Jukema et al. (1995)	884	2	Pravastatin 40 mg	4.3	3.2	Placebo	4.3	4.4
Nissen et al. (2004)	654	1.5	Atorvastatin 80 mg	3.0	2.1	Pravastatin 40 mg	3.9	3.9
Armitage et al. (2010)	12,064	6.7	Simvastatin 80 mg	2.5	2.2	Simvastatin 20 mg	2.5	2.5
Rossebø et al. (2008)	1873	2	Ezetimibe 10 mg + Simvastatin 40 mg	3.6	1.7	Placebo	3.6	3.5
Baigent et al. (2011)	9,270	4.9	Ezetimibe 10 mg + Simvastatin 20 mg	2.8	1.9	Placebo	2.8	2.7
Amarenco et al. (2006)	4,731	4.9	Atorvastatin 80 mg	3.4	1.9	Placebo	3.5	3.3
LaRosa et al. (2005)	10,001	4.9	Atorvastatin 80 mg	2.5	2.0	Atorvastatin 10 mg	2.5	2.6
Shepherd et al. (1995)	6,595	4.9	Pravastatin 40 mg	5.0	3.7	Placebo	5.0	4.9

LDL-C, low-density lipoprotein cholesterol.

**FIGURE 2:**
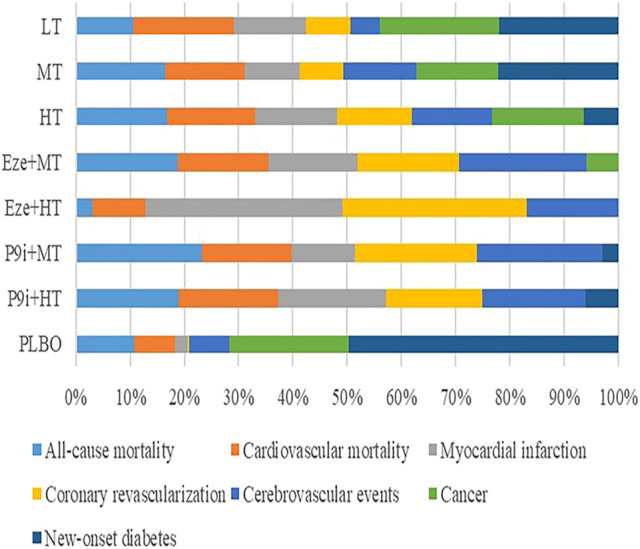
Rankogram showing the probability analysis of clinical outcomes included among the interventions. LT, low-intensity statins; MT, moderate-intensity statins; HT, high-intensity statins; Eze+MT, ezetimibe + moderate-intensity statins; Eze+HT, ezetimibe + high-intensity statins; P9i+MT, PCSK9i + moderate-intensity statins; P9i+HT, PCSK9i + high-intensity statins; PLBO, placebo.

The Cochrane Risk of Bias Tool was used to examine the quality of each selected trial, and 74.8, 16.8, and 8.4% of the studies were considered as low risk, unclear risk, and high risk, respectively. The risk of studies was mainly caused by blind assignments and other bias during the trial design (Supplementary Figure S1). The funnel plots did not show publication bias (Supplementary Figures S2, S3). All-cause mortality and coronary revascularization showed statistical heterogeneity, with I^2^ values of 57 and 63%, respectively. There were no inconsistencies in direct and indirect comparisons among all outcomes. The network meta-analysis of the clinical outcomes is summarized in Table 2, and the results of the SUCRA rank on all the clinical outcomes is presented in Figure 2.

**TABLE 2:** Network meta-analysis for clinical outcomes included.

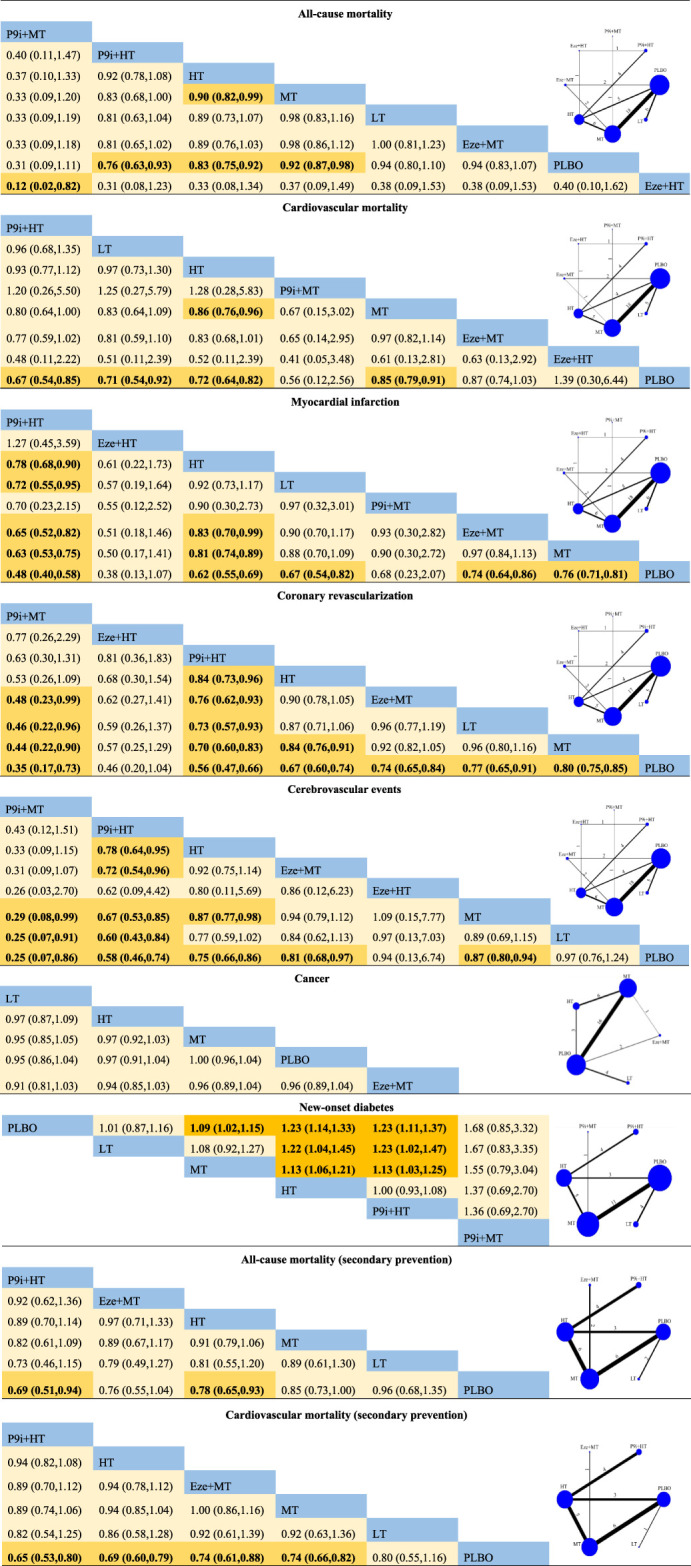

Each area represents the value of RR and 95% CI. The blue area represents interventions; the orange area and bolded values represent statistically meaningful at the 0.05 significance level. The number perpendicular to the line between the two interventions represents the number of studies included. P9i + HT, PCSK9i + high-intensity statins; P9i + MT, PCSK9i + moderate-intensity statins; Eze + HT, ezetimibe + high-intensity statins; Eze + MT, ezetimibe + moderate-intensity statins; HT, high-intensity statins; MT, moderate-intensity statins; LT, low-intensity statins; PLBO, placebo.

### Primary Outcomes

Forty-six and 42 studies were evaluated for the risk of all-cause mortality and cardiovascular mortality, with 283,867 and 281,305 participants, respectively. The meta-analysis showed that the more intensive treatment could significantly reduce all-cause mortality (RR 0.91, 95% CI 0.88–0.95) and cardiovascular mortality (RR 0.89, 95% CI 0.86–0.92) compared with the less intensive treatment (Supplementary Figures S4, S5).

**FIGURE 3:**
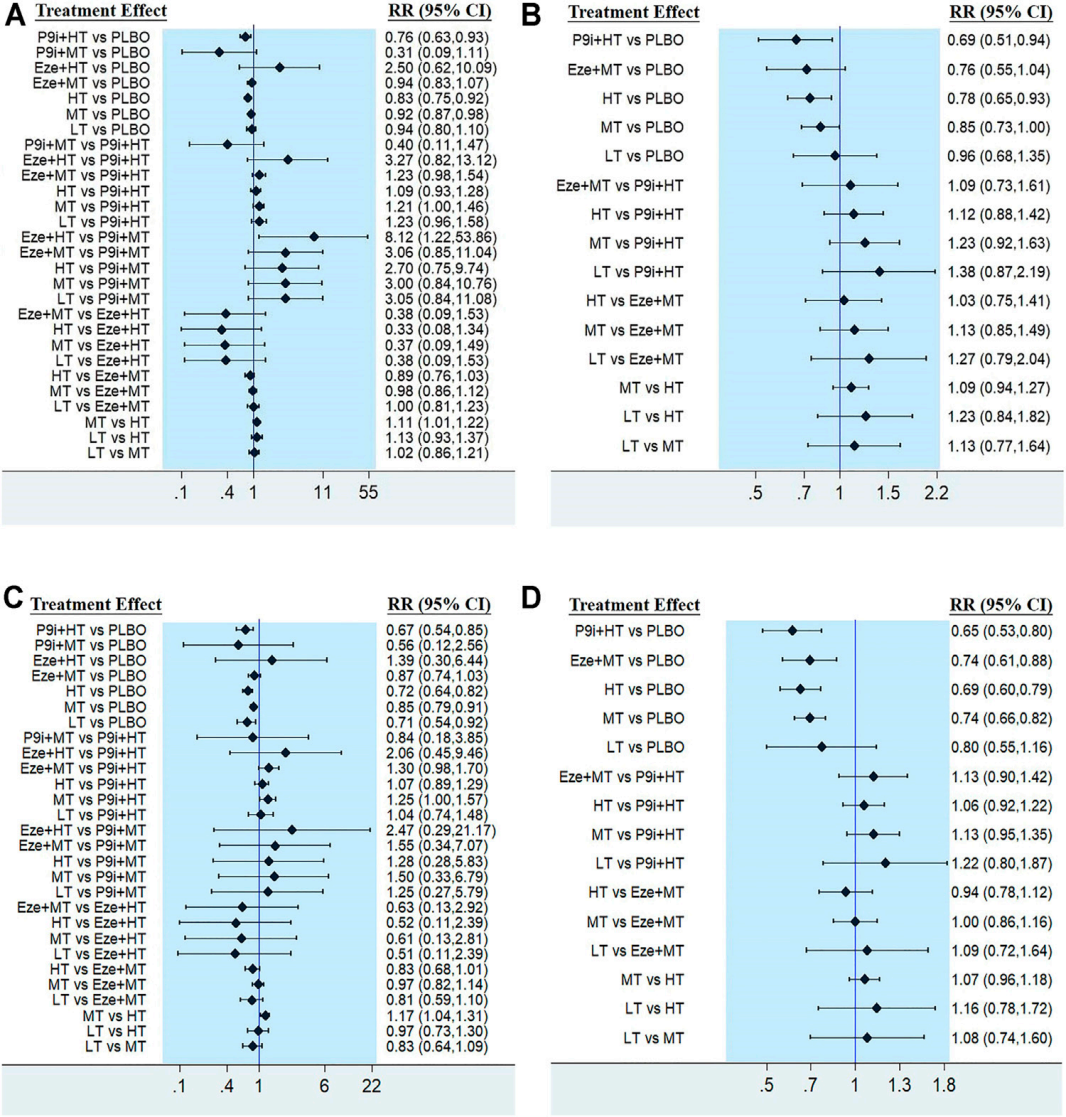
Forest plots showing comparison among interventions for **(A)** All-cause mortality, **(B)** All-cause mortality of secondary prevention, **(C)** Cardiovascular mortality, **(D)** Cardiovascular mortality of secondary prevention. PLBO, placebo; P9i+HT, PCSK9i + high-intensity statins; P9i+MT, PCSK9i + moderate-intensity statins; Eze+HT, ezetimibe + high-intensity statins; Eze+MT, ezetimibe + moderate-intensity statins; HT, high-intensity statins; MT, moderate-intensity statins; LT, low-intensity statins; RR, risk ratios; CI, confidence intervals.

The network plots of seven interventions for all-cause mortality are presented in Figure 3A. Compared with PLBO, the network meta-analysis showed that P9i + HT (RR 0.76, 95% CI 0.63–0.93), HT (RR 0.83, 95% CI 0.75–0.92), and MT (RR 0.92, 95% CI 0.87–0.98) significantly reduced all-cause mortality. HT associated with a 10% (RR 0.90, 95%CI 0.82–0.99) risk reduction in all-cause mortality compared with MT. The network meta-analysis of secondary prevention showed that P9i + HT associated with a 31% risk reduction (RR 0.69, 95% CI 0.51–0.94), whereas HT associated with a 22% risk reduction (RR 0.78, 95% CI 0.65–0.93) compared with PLBO (Figure 3B). P9i + HT and P9i + MT ranked as the most effective treatments in reducing all-cause mortality for secondary prevention and all populations, respectively (SUCRA 86.0 and 95.3%).

The efficacy of seven interventions in reducing cardiovascular mortality was similar to that of all-cause mortality (Figure 3C). P9i + HT, LT, HT, and MT associated with 33% (RR 0.67, 95% CI 0.54–0.85), 29% (RR 0.71, 95% CI 0.54–0.92), 28% (RR 0.72, 95% CI 0.64–0.82), and 15% (RR 0.85, 95% CI 0.79–0.91) risk reduction in cardiovascular mortality compared with PLBO, respectively. Moreover, HT reduced the risk of cardiovascular mortality by 14% (RR 0.86, 95% CI 0.76–0.96) compared with MT. The network meta-analysis of secondary prevention showed that P9i + HT reduced the risk of cardiovascular mortality by 35% (RR 0.65, 95% CI 0.53–0.80), HT by 31% (RR 0.69, 95% CI 0.60–0.79), Eze + MT by 26% (RR0.74, 95% CI 0.61–0.88), and MT by 26% (RR 0.74, 95% CI 0.66–0.82) compared with PLBO (Figure 3D), with P9i + HT ranking as the most effective drug in all population and secondary prevention (SUCRA 79.3 and 88.0%).

### Secondary Outcomes

Traditional meta-analysis showed that the more intensive treatment could reduce the risk of MI, coronary revascularization, and cerebrovascular events compared with the less intensive treatment (Supplementary Figures S6–S8).

In total, 283,867 participants from 46 studies provided the MI data in the network meta-analysis of seven interventions. Compared with PLBO, P9i + HT (RR 0.48, 95% CI 0.40–0.58), HT (RR 0.62, 95% CI 0.55–0.69), LT (RR 0.67, 95% CI 0.54–0.82), Eze + MT (RR 0.74, 95% RR 0.64–0.86), and MT (RR 0.76, 95% CI 0.71–0.81) could effectively reduce the risk of MI. HT associated with lower MI risk than MT (RR 0.81, 95% CI 0.74–0.89) and Eze + MT (RR 0.83, 95% CI 0.79–0.99). The risk of MI in patients who received P9i + HT was significantly reduced compared with those who received Eze + MT (RR 0.65, 95% CI 0.52–0.82) and different intensity statins (HT, RR 0.78, 95% CI 0.68–0.90; MT, RR 0.63, 95% CI 0.53–0.75; LT, RR 0.72, 95% CI 0.55–0.95) (Supplementary Figure S9). Compared with other interventions, P9i + HT ranked first in reducing MI risk (SUCRA 86.2%).

There were 276,077 participants from 42 studies in the network meta-analysis of seven interventions, which included coronary revascularization events. Compared with PLBO, P9i + MT reduced the relative risk of coronary revascularization by 65% (RR 0.35, 95% CI 0.17–0.73), P9i + HT by 44% (RR 0.56, 95% CI 0.47–0.66), HT by 33% (RR 0.67, 95% CI 0.64–0.83), Eze + MT by 26% (RR 0.74, 95% CI 0.65–0.84), LT by 23% (RR 0.77, 95% CI 0.65–0.91), and MT by 20% (RR 0.80, 95% CI 0.75–0.85) (Figure). P9i + MT caused a significant reduction in the risk of coronary revascularization by 52% (RR 0.48, 95% CI 0.23–0.99), by 54% (RR 0.46, 95% CI 0.22–0.96), and by 56% (RR 0.44, 95% CI 0.22–0.90), respectively, compared with Eze + MT, LT, and MT. Compared with HT, P9i + HT associated with 16% risk reduction in coronary revascularization (Supplementary Figure S10). Among the seven interventions, P9i + MT yielded the highest SUCRA rank (92.3%) in reducing coronary revascularization events.

Forty-four studies, which included 281,305 participants provided the cerebrovascular event dates. The network meta-analysis showed that P9i + MT (RR 0.25, 95% CI 0.07–0.86), P9i + HT (RR 0.58, 95% CI 0.46–0.74), HT (RR 0.75, 95% CI 0.66–0.86), Eze + HT (RR 0.81, 95% CI 0.66–0.86), and MT (RR 0.87, 95% CI 0.80–0.94) had a lower risk of cerebrovascular events than PLBO (Figure). P9i + HT associated with higher benefits of cerebrovascular events compared with Eze + MT (RR 0.72, 95% CI 0.54–0.96) and different intensity statins (HT, RR 0.78, 95% CI 0.64–0.95; MT, RR 0.67, 95% CI 0.53–0.85; LT, RR 0.60, 0.43–0.84). HT associated with a lower risk of cerebrovascular events than MT (RR 0.87, 95% CI 0.77–0.98) (Supplementary Figure S11). Overall, P9i + MT had the highest probability of ranked best (SUCRA 95.1%).

### Other Outcomes

The cancer data was provided by 216,665 participants in 32 studies. The traditional meta-analysis described no statistical difference in the risk of cancer on lipid-lowering drugs (RR 0.99, 95% CI 0.96–1.03, *p* = 0.70, I^2^ = 1%) (Supplementary Figure S12). In the network meta-analysis, there was no evidence that the seven interventions associated with the occurrence of cancer (Supplementary Figure S13).

**FIGURE 4:**
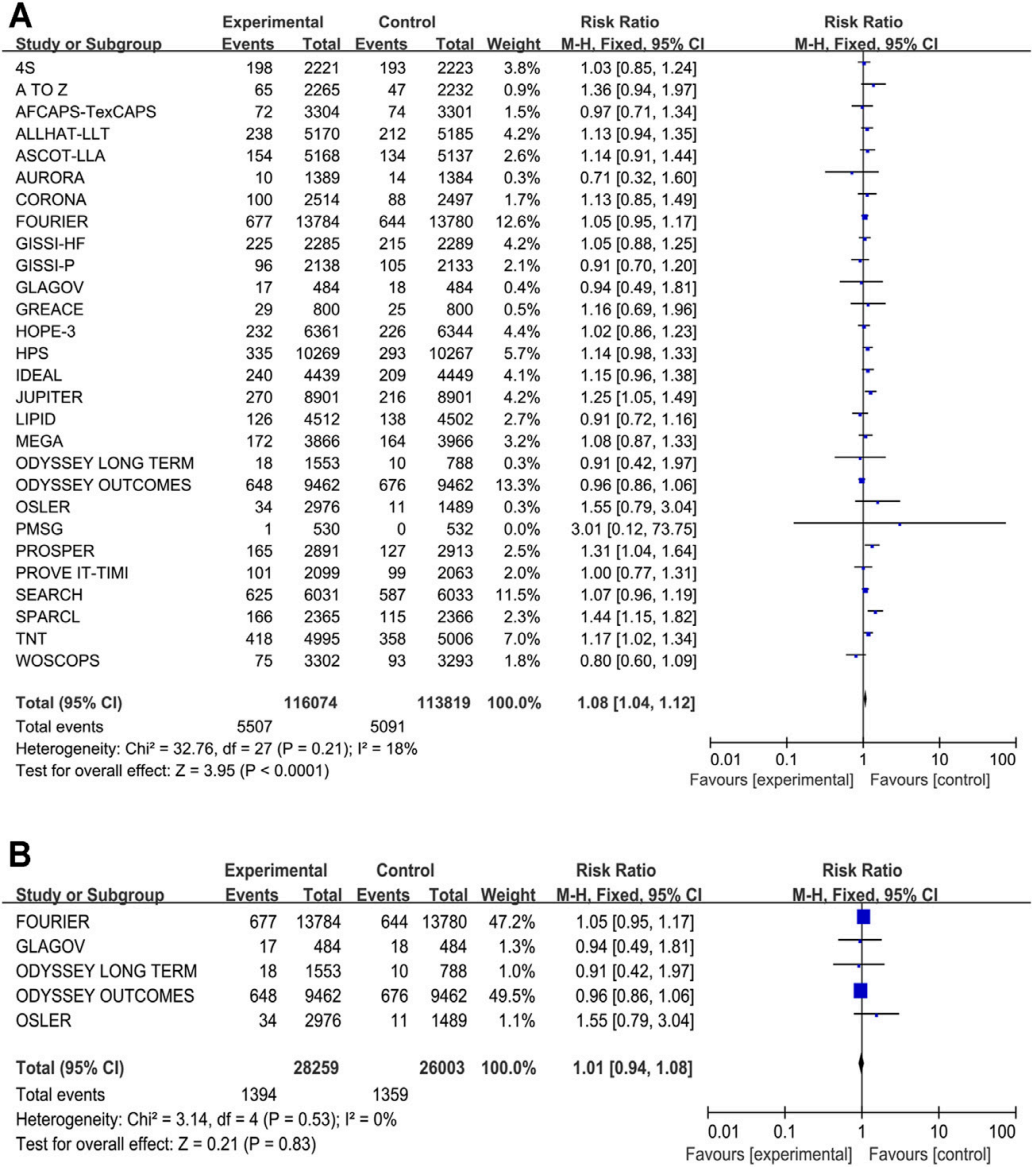
Meta-analysis of new-onset diabetes. **(A)** Forest plots of new-onset diabetes between the more intensive treatment and the less intensive treatment. **(B)** Subgroup meta-analysis of new-onset diabetes between PCSK9 inhibitors and statins. CI, confidence interval.

Twenty-eight studies, which included 229,893 participants, provided the data new-onset diabetes data. The traditional meta-analysis showed that the more-intensive lipid-lowering drugs associated with a higher risk of new-onset diabetes compared with the less-intensive lipid-lowering drugs conducted by our meta-analysis (RR 1.08, 95% CI 1.04–1.12, *p* < 0.001, I^2^ = 18%) (Figure 4A). When participants received P9i + HT (RR 1.23, 95% CI 1.11–1.37), HT (RR 1.23, 95% CI 1.14–1.33), or MT (RR 1.09, 95% CI 1.02–1.05), the likelihood of new-onset diabetes was significantly higher than that in participants who received PLBO (Supplementary Figure S14). P9i + MT might have been related to the occurrence of diabetes in terms of the SUCRA rank (11.8%). However, our subgroup analysis showed that there was no significant difference between PCSK9i added to background statins and moderate-to-high intensity statins (RR 1.01, 95% CI 0.94–1.08, *p* = 0.83) (Figure 4B).

## Discussion

In the present network meta-analysis, we report that PCSK9i added to background moderate-to-high intensity statins was the most effective lipid-lowering drug in reducing the risk of mortality and cardiovascular-related events. P9i + HT associated with 24% and 33% risk reduction in all-cause and cardiovascular mortality compared with PLBO in all studies, respectively. These results agreed with the analysis of secondary prevention in our network meta-analysis. Whether PCSK9i can reduce mortality, however, is controversial (Schmidt et al., 2020). Our findings showed that PCSK9i added to background high-intensity statins was associated with a reduced risk of all-cause mortality and cardiovascular mortality. However, PCSK9i did not show the benefit of reducing mortality even in the reliable FOURIER (Sabatine et al., 2017) and ODYSSEY OUTCOMES trial (Schwartz et al., 2018). Statins, the most used drug, have been reported to be effective in reducing mortality in multiple studies. However, in our study, statins were not superior to PCSK9i in reducing the risk of mortality. The reason for this result might have been due to the fact that the included studies had follow-up periods of at least 48 weeks because clinical benefits had been shown to be evident at approximately 1 year after drug use. This compensated for the shortcomings of the previous meta-analysis due to the short follow-up time, and we confirmed the beneficial effect of PCSK9i on mortality. This provides support for the wide clinical application of PCSK9i. In contrast, Diaz R et al. found from ODYSSEY OUTCOMES trial that the intensity of statin background was not associated with a reduced risk of MACE from alirocumab (Diaz et al., 2021). Similar LDL-C baseline levels in ODYSSEY OUTCOMES trial may contribute to this result, as higher baseline LDL-C levels were associated with a greater reduction in risk of MACE (Navarese et al., 2018). At the same time, alirocumab achieved a similar relative reduction in LDL-C levels in subgroups with different intensity statin background, leading to no difference in the risk of MACE among the groups from ODYSSEY OUTCOMES trial. The combination of statin and PCSK9i is necessary, as statin therapy increases both PCSK9 and LDL receptor levels. More trials are needed in the future to determine whether the reduced risk of clinical outcome associated with PCSK9i is related to background statin intensity.

A major safety issue with regard to the use of PCSK9i was whether lowering LDL-C levels could cause neurocognitive disorders. Ying et al. (Ying et al., 2021), recent EBBINGHAUS study (Gencer et al., 2020) as well as the CANTAB study (Janik et al., 2021), reported that PCSK9i had no impact on cognitive impairment. The OSLER study (Sabatine et al., 2015), which involved background moderate-intensity statin therapy, reported frequent neurocognitive adverse events. At the same time, PCSK9i has been reported in many meta-analysis as associated with neurocognitive disorder (Harvey et al., 2018; Gouverneur et al., 2021). However, Khan and Raccah et al. performed that evidence of neurocognitive adverse events was found during follow-up periods (Hirsh Raccah et al., 2021; Khan et al., 2017). In our network meta-analysis, we were unable to analyze the adverse neurological events due to the few studies that reported relevant events. Therefore, the follow-up time should be increased to evaluate the relationship between PCSK9i and neurological adverse events. Another safety issue is whether lipid-lowering drugs affect the health or clinical benefits of elderlies (75 years and older). Fortunately, lipid-lowering drugs treatment can be as effective in elderlies as they are in younger individuals (Collaboration, 2019; Gencer et al., 2020). Moreover, C-reactive protein (CRP) and lipoprotein(a) as risk factors for ASCVD. The CRP is an important inflammatory factor in the development of ASCVD, but there were no studies showing that PCSK9i affects CRP level (Ruscica et al., 2019). In the trials of PCSK9i, changes in the CRP level might have been caused by the background statins treatment. Lipoprotein(a) plays an independent risk factor for ASCVD. Lowering lipoprotein(a) 1.7 mmol/L could achieve similar cardiovascular benefits of reducing LDL-C levels by 1 mmol/L, but statins have not been shown to have a reduced lipoprotein(a) effect (Lamina and Kronenberg, 2019; Ruscica et al., 2021). PCSK9i, which co-downregulated lipoprotein(a) and LDL-C levels, has also been shown to obtain more clinical benefits (O'Donoghue et al., 2019). Unfortunately, the mechanism by which reduces lipoprotein(a) remains unknown.

Ezetimibe could further reduce LDL-C levels by 30% compared with statins alone. The IMPROVE-IT study presented the ezetimibe could improve cardiovascular outcomes and reduce the risk of adverse events (Cannon et al., 2015). However, there were no clinical benefits of ezetimibe added to background statins over statins alone. The reason for this result might be that ezetimibe was not widely used and the follow-up time was shorter than that of statins, such that its clinical benefits are not evident. Besides, the SEAS study showed that ezetimibe can increase the risk of cancer (Rossebø et al., 2008). No lipid-lowering drug was found to be associated with an increased risk of cancer in our analysis. Therefore, further studies are needed to verify the clinical benefits of ezetimibe.

In our network meta-analysis, we found that LT could effectively reduce the risk of cardiovascular mortality, MI, and coronary revascularization, and fortunately, it did not increase the risk of cancer and new-onset diabetes. LT had a higher probability of associating with lower MI risk than P9i + MT and Eze + MT in our network meta-analysis. It was an indisputable fact that PCSK9i and ezetimibe could significantly reduce LDL-C levels than LT. However, a recent study reported that ApoB was a more accurate marker of myocardial infarction than LDL-C (Johannesen et al., 2021). Therefore, exclusive use of the degree of lowered LDL-C level is insufficient to evaluate the risk of MI. This clinical outcome might have been related to the lifestyle and baseline body weight of the individuals. Moreover, the MEGA trial stated that low-intensity statins could achieve the same benefits as high-intensity statins in primary prevention in Japanese individuals (Nakamura et al., 2006), suggesting that different sensitivities to statin therapy might exists in different regions and races. Patients at risk of ASCVD are often initially treated with moderate-intensity statins. As such, the results of our network meta-analysis provide a basis for lowering blood lipids with LT for initial therapy.

We found that the risk of new-onset diabetes was associated with P9i + HT, HT, and MT. The reason for this result might be related to LDL-C level. Da Dalt et al. showed that PCSK9 deficiency could limit insulin secretion, leading to hyperglycemia (Da Dalt et al., 2019). Moreover, a recent study reported that depression caused an increase in PCSK9 level, which lead to insulin resistance (Macchi et al., 2020). However, Khan et al. (Khan et al., 2019), as well as a subanalysis from FOURIER trial (Sabatine et al., 2017), reported that PCSK9i, the most effective lipid-lowering drug, did not increase the risk of diabetes. Chiu et al. also stated that PCSK9i was not associated with the risk of diabetes (Chiu et al., 2020). Besides, PCSK9i added to background statins could increase the risk of new-onset diabetes, which might have been due to statin use. Our analysis confirmed that PCSK9i did not increase the additional risk of new-onset diabetes compared with statins, which had a conflicting conclusions of JUPITER (Ridker et al., 2008) and WOSCOPS (Shepherd et al., 1995) trials on the involvement of statins in the risk of diabetes. We found that HT was more likely to induce diabetes than low-to-moderate intensity statins. Wang et al. demonstrated that more intensive statins might increase the risk of new-onset diabetes by 18% (Wang et al., 2017). In the included studies, the high-intensity statins were atorvastatin (≥40 mg), rosuvastatin (≥20 mg), and simvastatin (≥80 mg) (Stone et al., 2014), suggesting that these high-intensity statins might be correlated with new-onset diabetes. However, the specific mechanism by which statins cause diabetes is still unclear. Therefore, in the long-term use of statins, blood glucose monitoring should be carried out. In addition, the mechanism of its occurrence should be thoroughly explored, and whether this situation can be improved by the use of combination drugs.

Of course, statins are often used in combination with other non-statins, in addition to PCSK9i and ezetimibe, which are commonly used. Recent network analysis performed that non-statin drugs, such as omega-3 fatty acids, bile acid sequestrants, and fibrates, when combined with statins, did not have positive effects on cardiovascular outcomes; instead, they increased the incidence of adverse events (Hoang and Kim, 2020; Kim et al., 2020). The demands for non-statin lipid-lowering drugs have increased due to statin-related muscle symptoms, hepatobiliary side-effects, and familial hypercholesterolemia. Inclisiran, a siRNA targeting PCSK9, can reduce LDL-C levels and is currently in the rapid development stage (Khan et al., 2020). Evinacumab is an anti-ANGPTL3 monoclonal antibody, which has been used in the treatment of familial hypercholesterolemia (Raal et al., 2020). As many lipid-lowering drugs are gradually introduced into use, we should pay more attention to the long-term efficacy and safety of these drugs, and their ability to treat or halt the occurrence of adverse events.

We reviewed studies of PCSK9i and found that the trials on the combined use of PCSK9i and ezetimibe were absent. The guidelines showed that if maximally tolerant statins and ezetimibe could not achieve target LDL-C levels, then PCSK9i was recommended for use. However, there were no studies on the combined use of PCSK9i and ezetimibe with or without statins. GAUSS-1, GAUSS-2 and GAUSS-3 trials showed that PCSK9i could effectively reduce the risk of muscle adverse events in patients with statin intolerance compared with ezetimibe (Sullivan et al., 2012; Stroes et al., 2014; Nissen et al., 2016). Although the price of PCSK9i is presently higher than that of other drugs and less cost-effectiveness in general patients, the beneficial effects of these inhibitors are well documented, especially in patients with statin intolerance and familial hypercholesterolemia (Azari et al., 2020). In the future, the price of PCSK9i should be adjusted within the acceptable range of patients, which will help to bring greater cost-effectiveness and more clinical benefits.

The advantages of this network meta-analysis are that statins were divided into different intensities and compared with the commonly lipid-lowering drugs. Besides, the included studies were randomized controlled trials, which enhanced the reliability of the results. At the same time, our analysis provides evidence to support the ability of PCSK9i to reduce mortality. However, there are several intrinsic limitations. Firstly, our network meta-analysis was unable to assess primary prevention in isolation because there are not adequate studies using PCSK9i and ezetimibe in primary prevention, and the definition of primary prevention is different across studies (Naci et al., 2013; Yebyo et al., 2019), which can affect the results in this network meta-analysis. Secondly, we only analyzed all-cause mortality and cardiovascular mortality in secondary prevention, as traditional meta-analysis has confirmed the positive effects of lipid-lowering therapy on MACE. Thirdly, statins were divided into three intensities but there was no further analysis of the different types of statins that were involved. Besides, we could not analyze these endpoint events, as the adverse events, such as metabolic disorder syndrome and cognitive disorders, were only reported in few studies.

## Conclusion

In summary, the findings of our network meta-analysis have important clinical implications. We found that 1) more intensive treatment compared with less intensive treatment was associated with a greater risk reduction in all-cause mortality, MACE, and coronary revascularization; 2) PCSK9i added to background statins was ranked as the most effective treatment in reducing these outcomes, and PCSK9i did not increase the additional risk of new-onset diabetes; 3) LT could be recommended as the initial therapy; 4) the beneficial effects of ezetimibe on clinical outcomes were not superior to those of statins; and 5) clinicians should pay attention to the risk of new-onset diabetes when using moderate-to-high intensity statins. Lastly, lipid-lowering drugs were not associated with the development of cancer.

## Data Availability

The original contributions presented in the study are included in the article/Supplementary Material, further inquiries can be directed to the corresponding authors.
