# Supplementary material for: Efficacy and Safety of Lipid-Lowering Drugs of Different Intensity on Clinical Outcomes: A Systematic Review and Network Meta-Analysis
Source: Front Pharmacol. 2021 Oct 21;12:713007. doi: 10.3389/fphar.2021.713007 (PMC8567017; doi:10.3389/fphar.2021.713007)
Supplement: Supplementary file 1 [file DataSheet1.docx]

Efficacy and Safety of Lipid-lowering Drugs of Different Intensity on Clinical Outcomes: a Systematic Review and Network Meta-analysis

Wenrui Ma, Qinyuan Pan, Defeng Pan, Tongda Xu, Hong Zhu, Dongye Li

**Supplementary material**

1. Supplementary figures

2. Supplementary tables


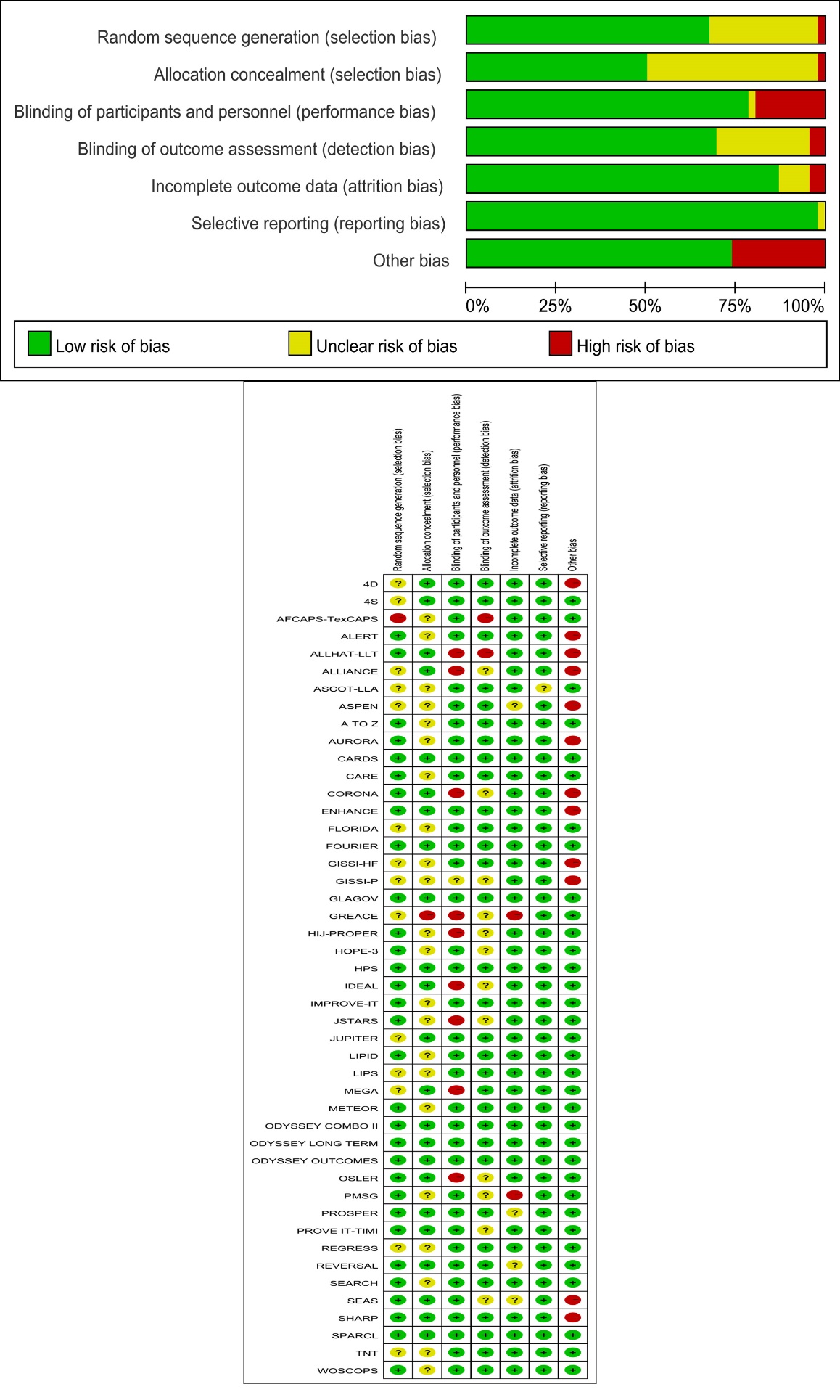


Figure S1. Risk of bias in the eligible RCTs followed the Cochrane risk of bias assessment tool.


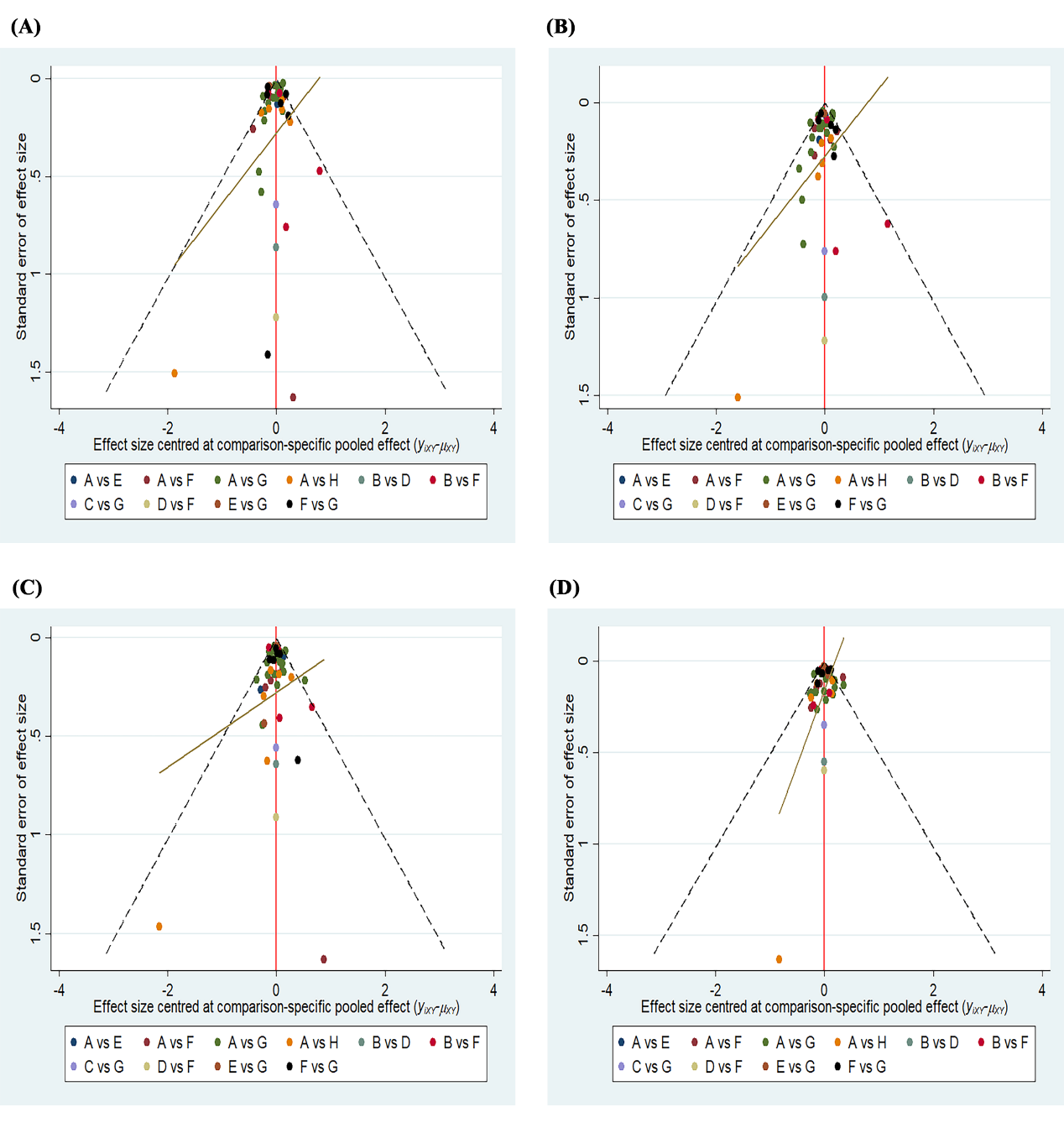


Figure S2. Funnel plot for publication bias on mortality and cardiovascular-related events. (A) All-cause mortality, (B) Cardiovascular mortality, (C) Myocardial infarction, (D) Coronary revascularization. A: placebo; B: PCSK9i + high-intensity statins; C: PCSK9i + moderate-intensity statins; D: ezetimibe + high-intensity statins; E: ezetimibe + moderate-intensity statins; F: high-intensity statins; G: moderate-intensity statins; H: low-intensity statins.


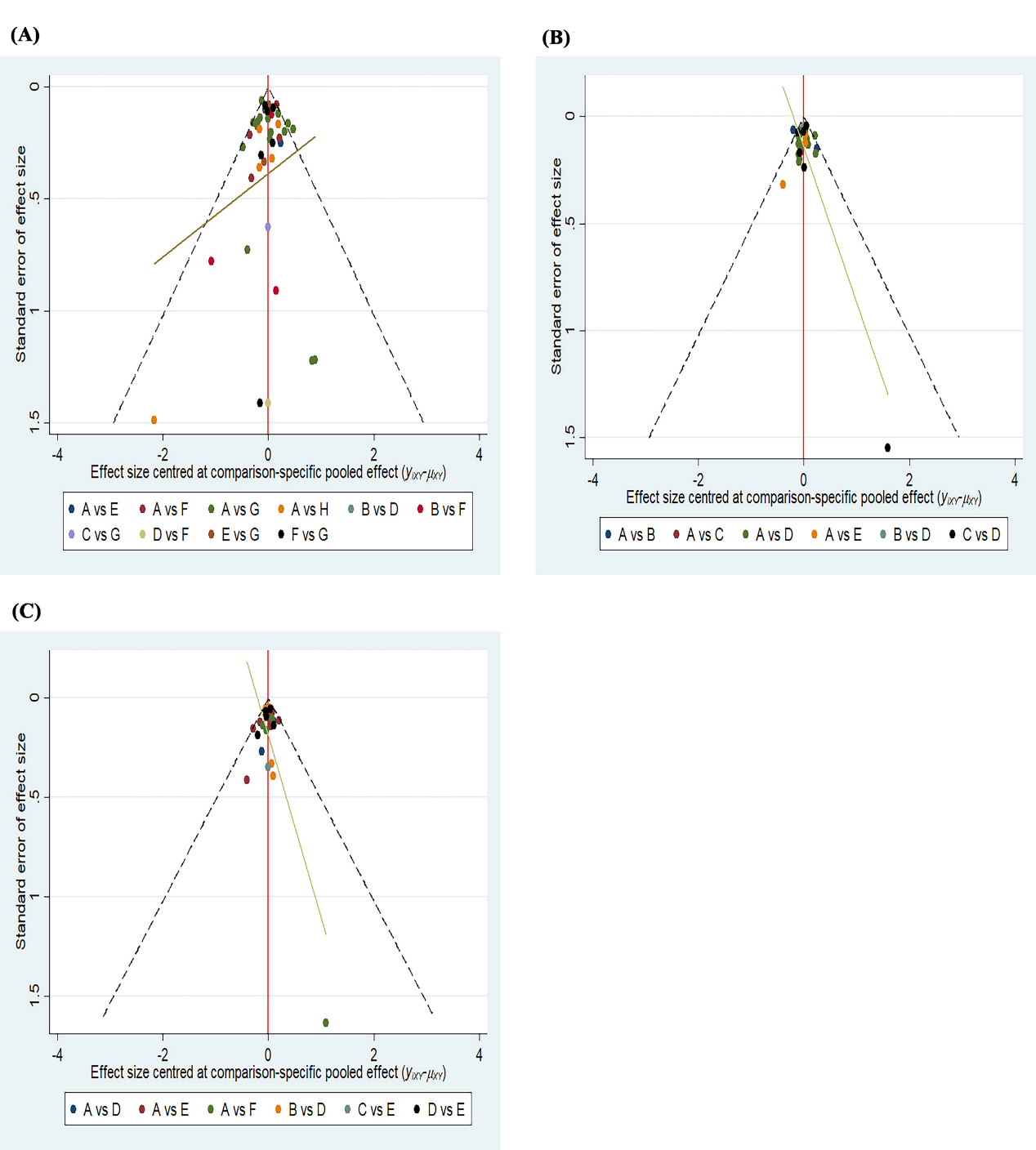


Figure S3. Funnel plot for publication bias on non-cardiovascular-related events. (A) Cerebrovascular events, (B) Cancer, (C) New-onset diabetes. A: placebo; B: PCSK9i + high-intensity statins; C: PCSK9i + moderate-intensity statins; D: ezetimibe + high-intensity statins; E: ezetimibe + moderate-intensity statins; F: high-intensity statins; G: moderate-intensity statins; H: low-intensity statins.


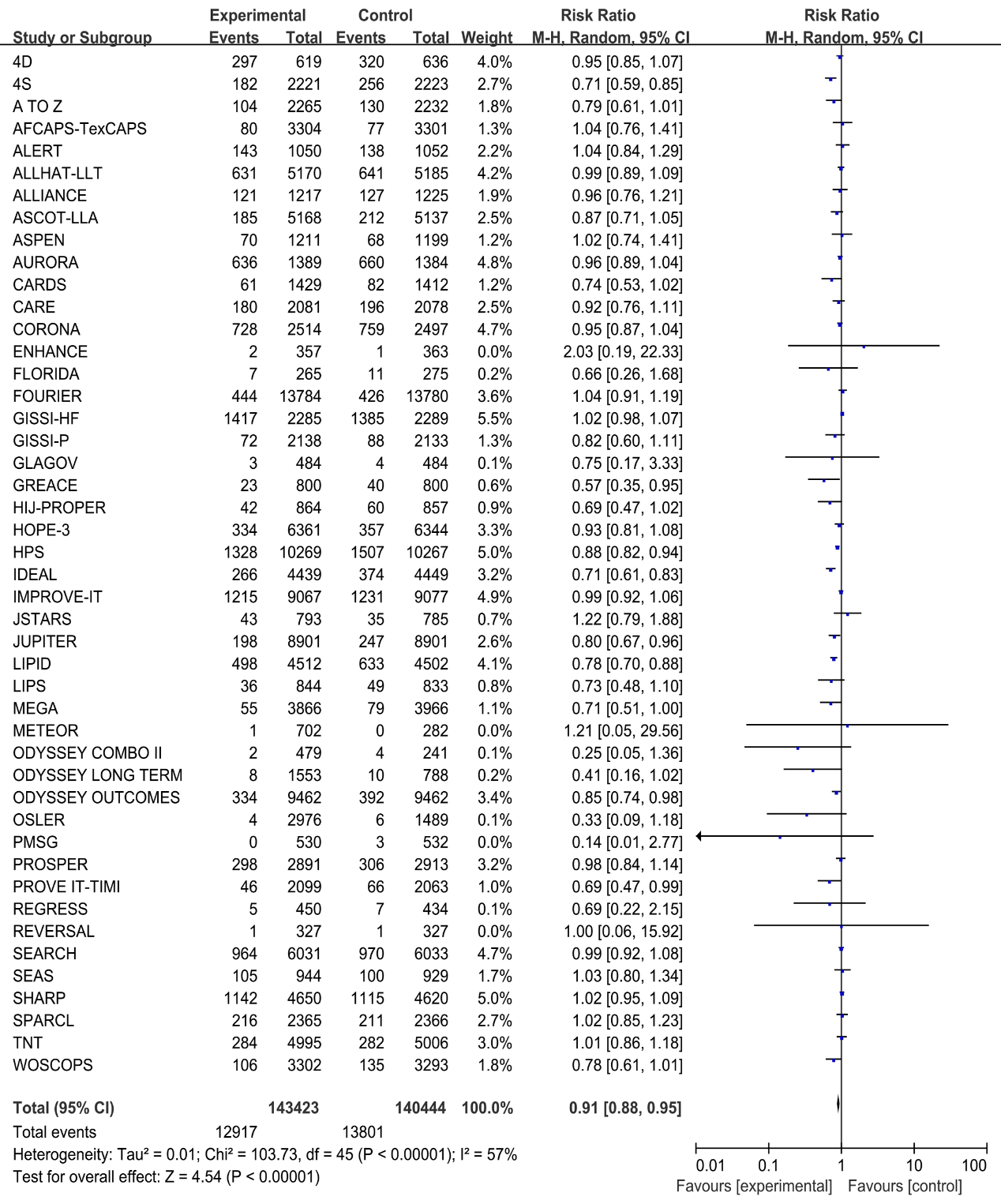


Figure S4. Forest plot of all-cause mortality in traditional meta-analysis.


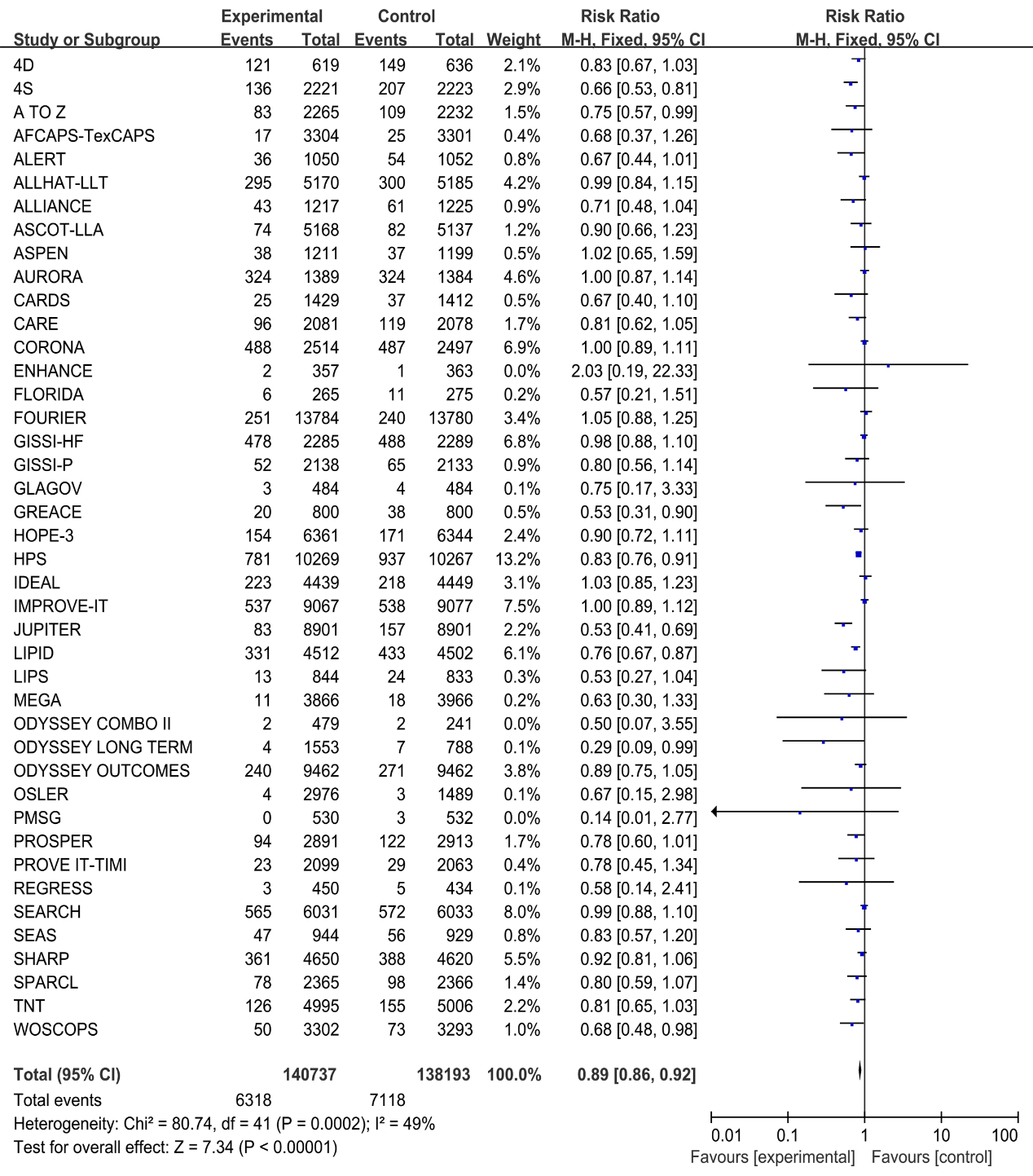


Figure S5. Forest plot of cardiovascular mortality in traditional meta-analysis.


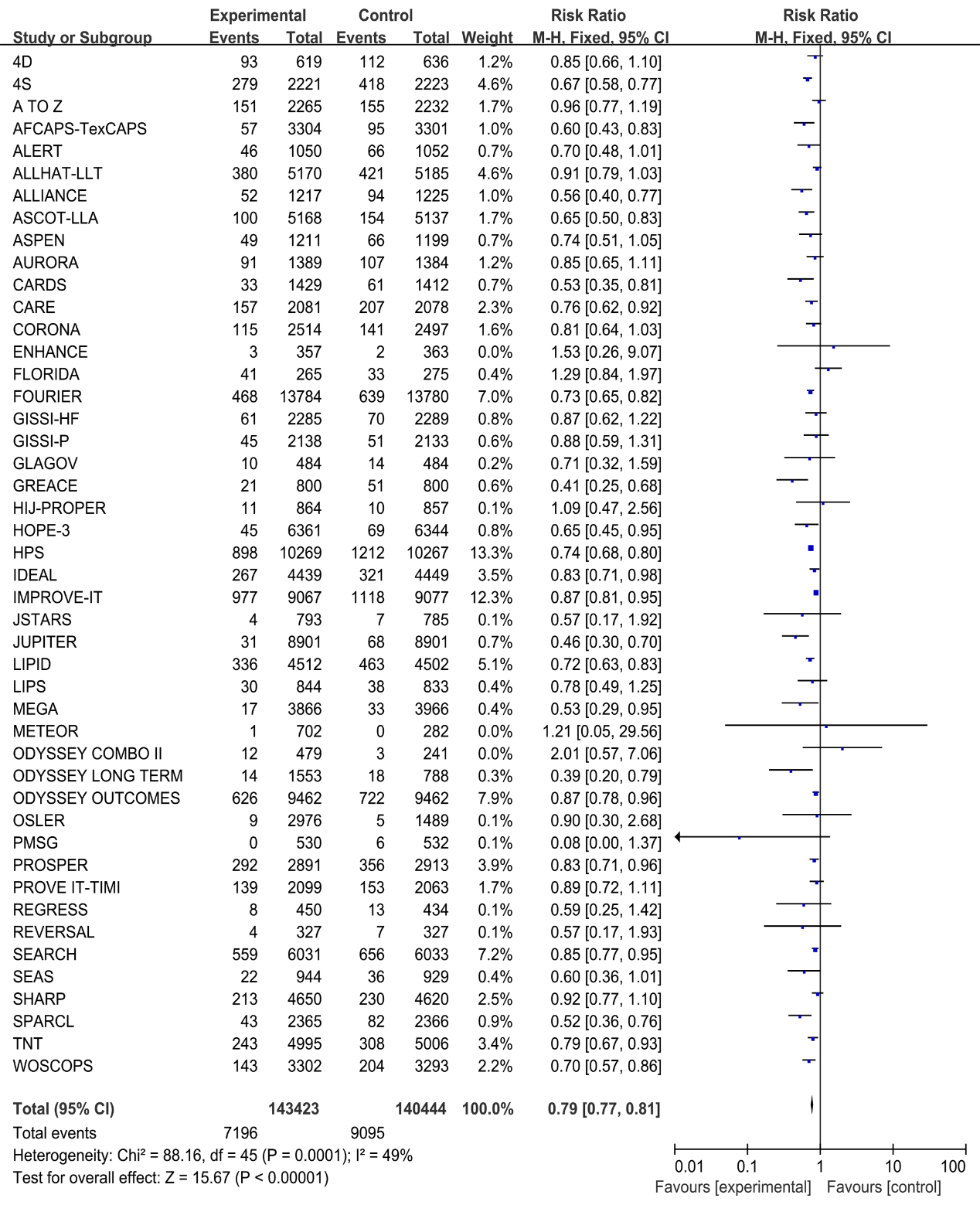


Figure S6. Forest plot of myocardial infarction in traditional meta-analysis.


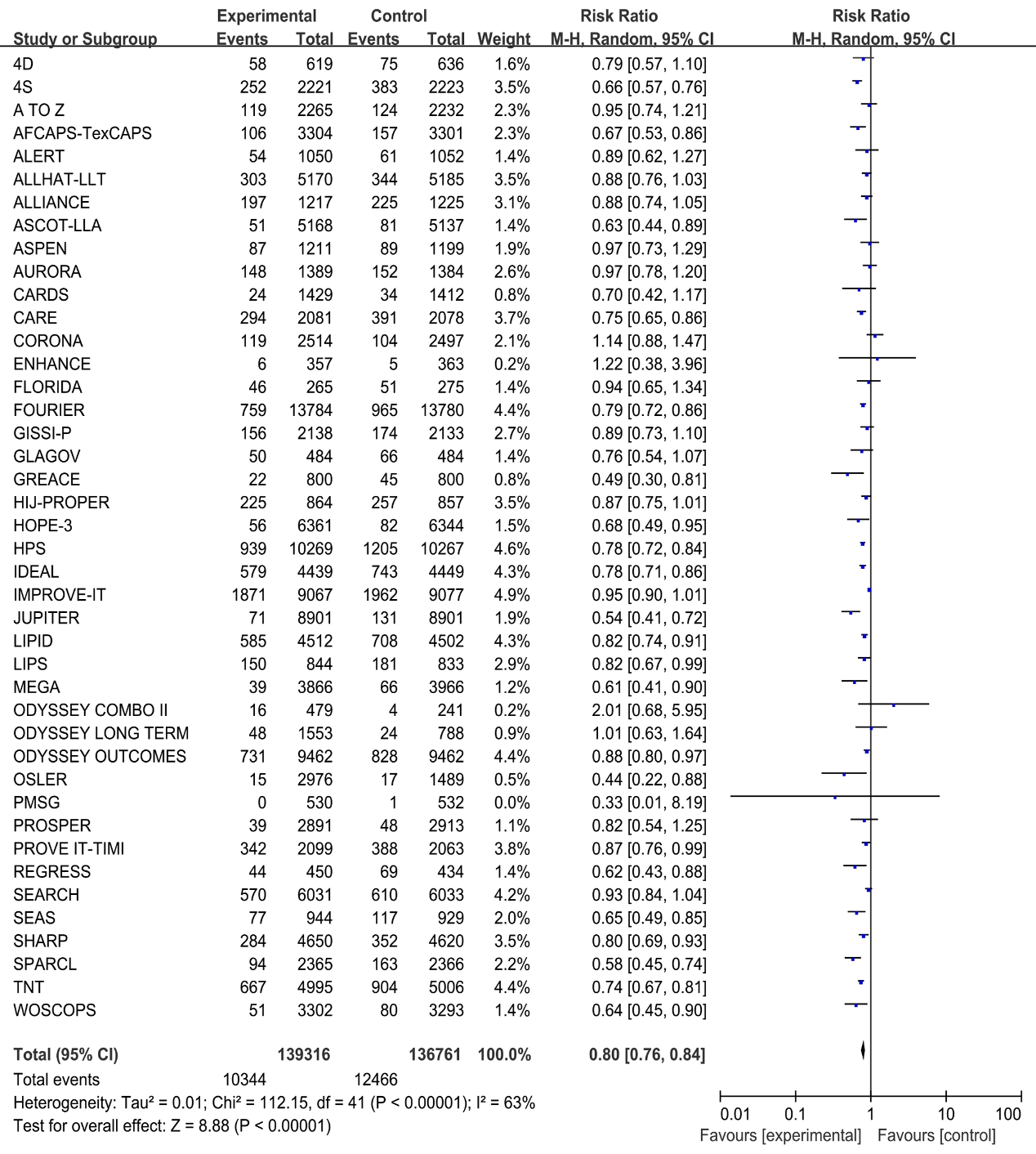


Figure S7. Forest plot of coronary revascularization in traditional meta-analysis.


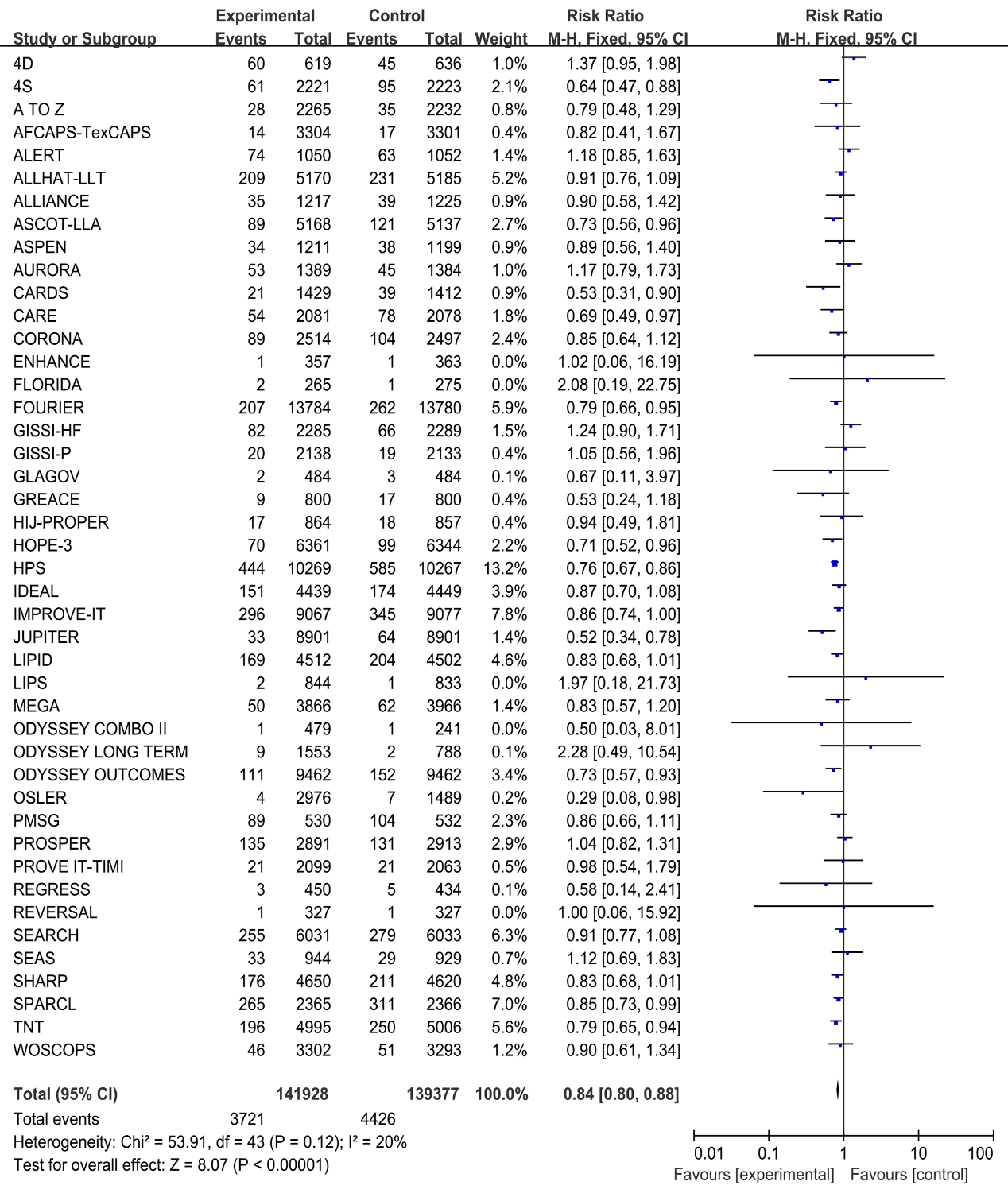


Figure S8. Forest plot of cerebrovascular events in traditional meta-analysis.


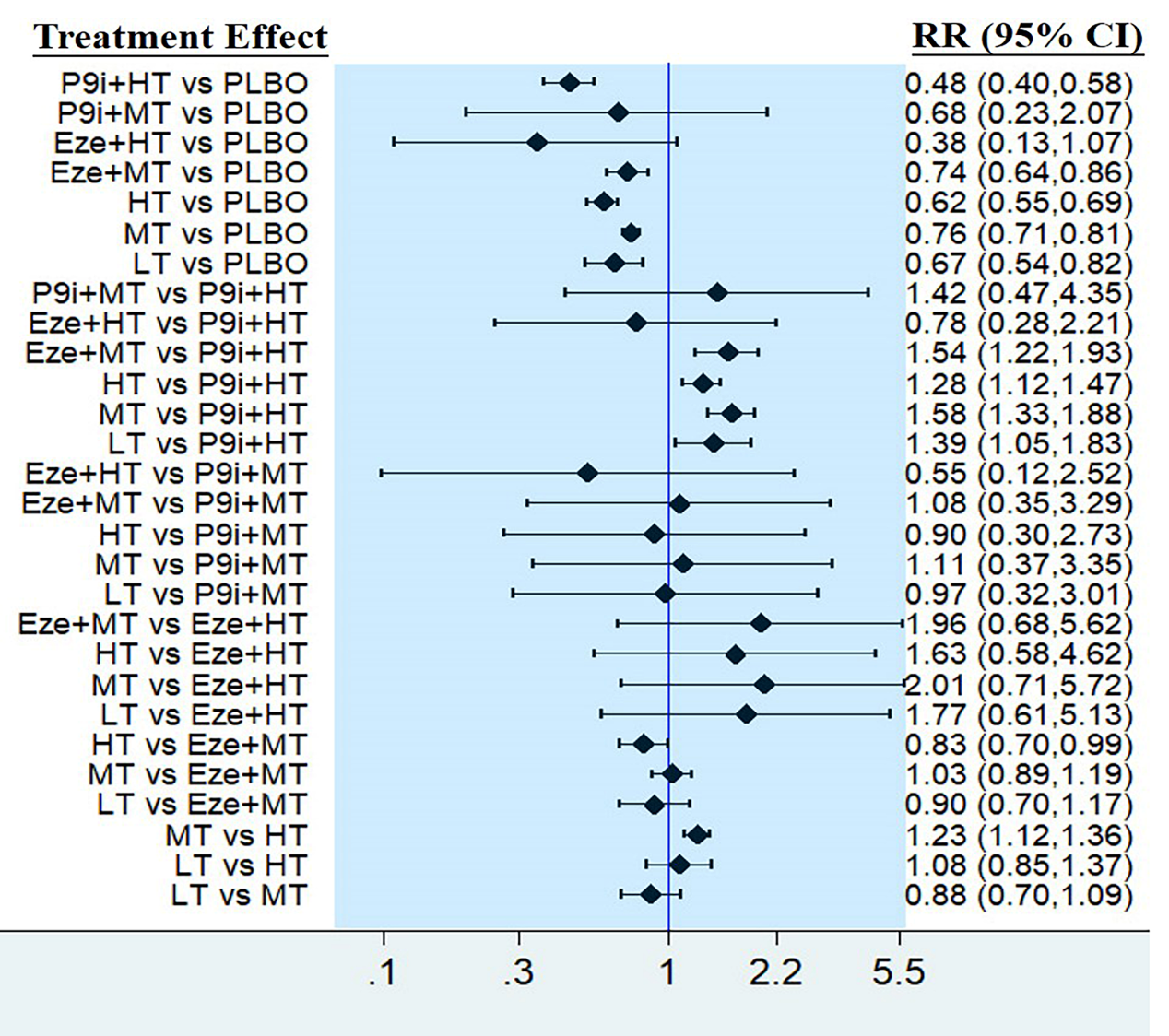


Figure S9. Forest plot of myocardial infarction in network meta-analysis.


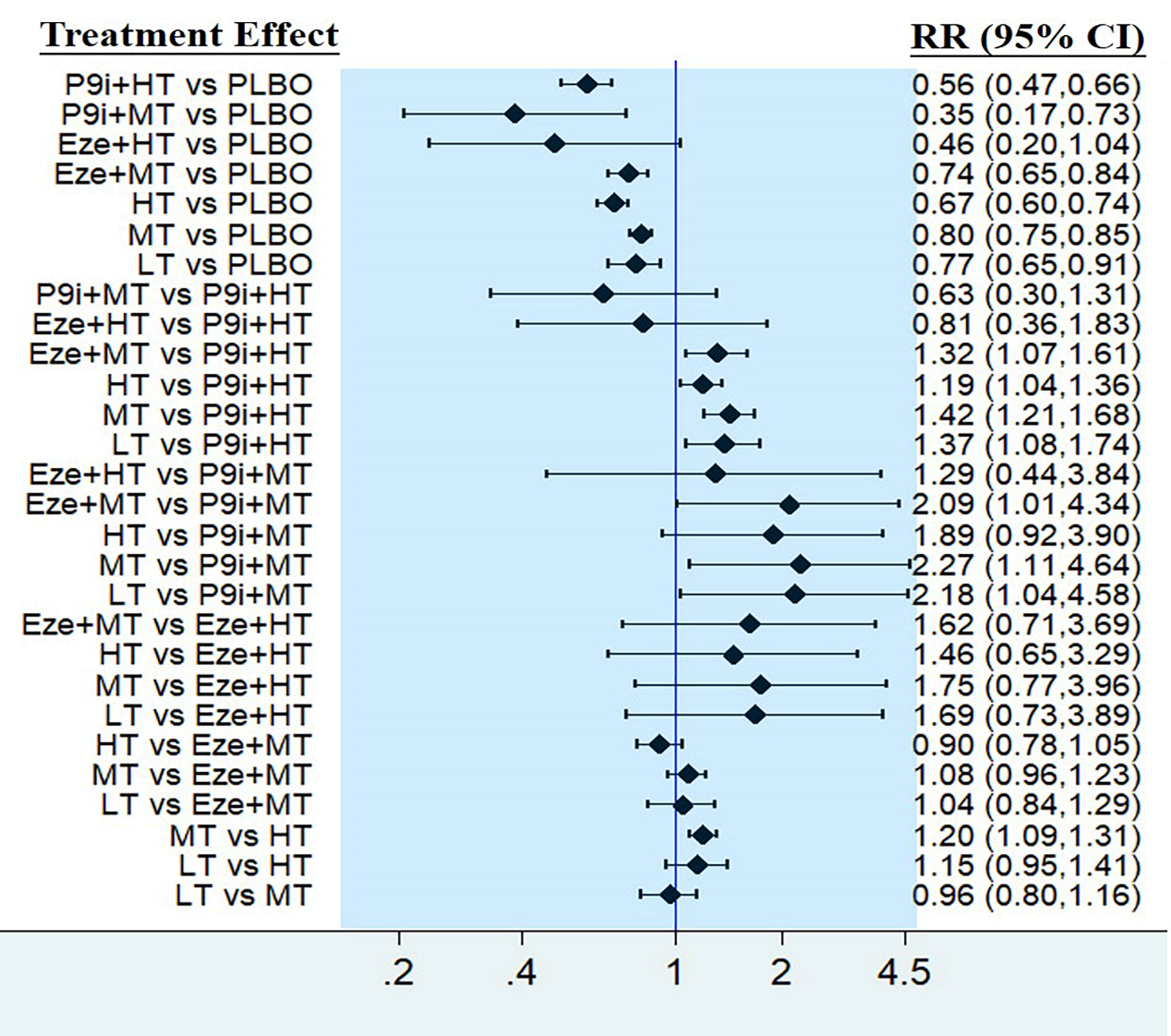


Figure S10. Forest plot of coronary revascularization in network meta-analysis.


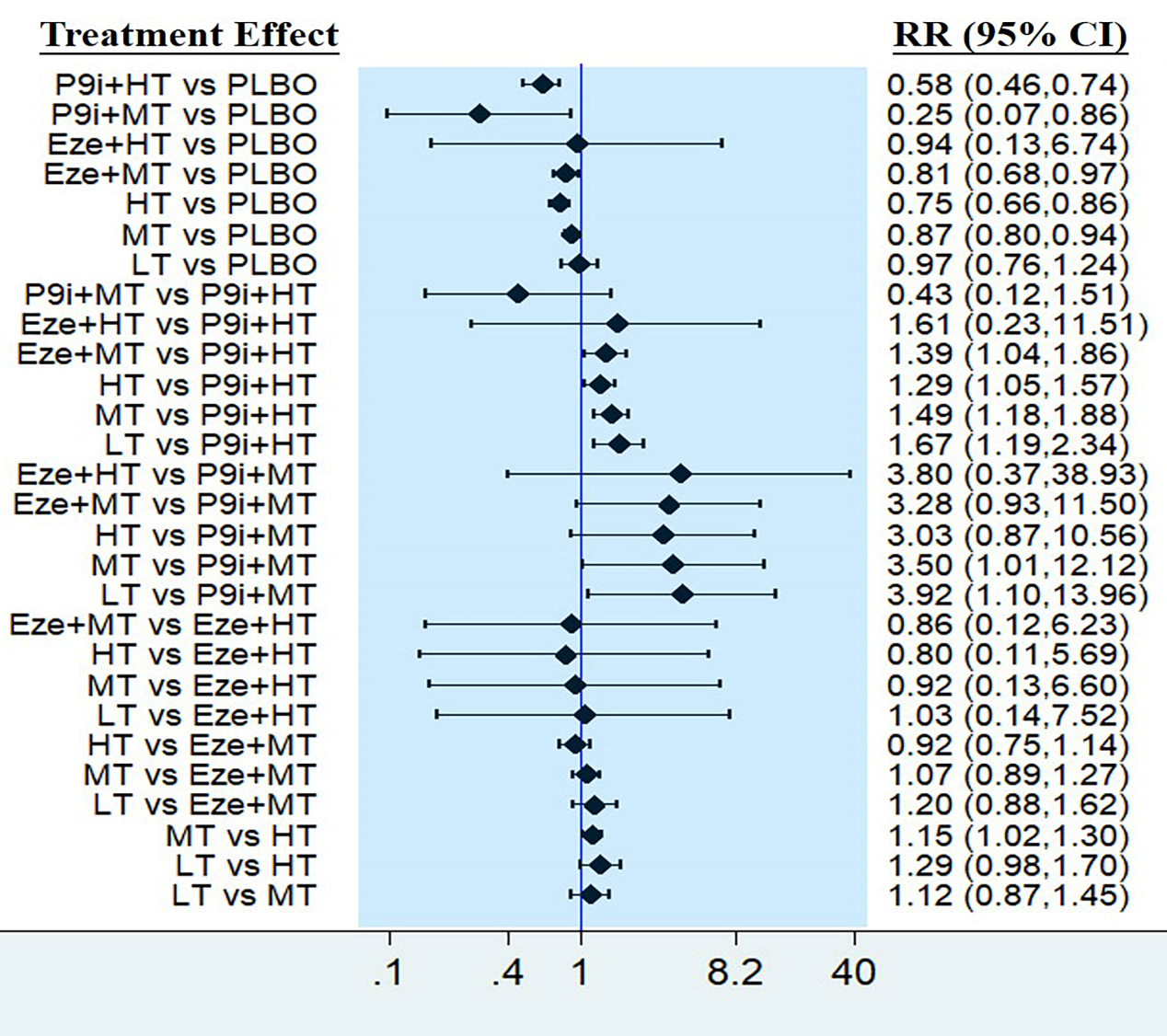


Figure S11. Forest plot of cerebrovascular events in network meta-analysis.


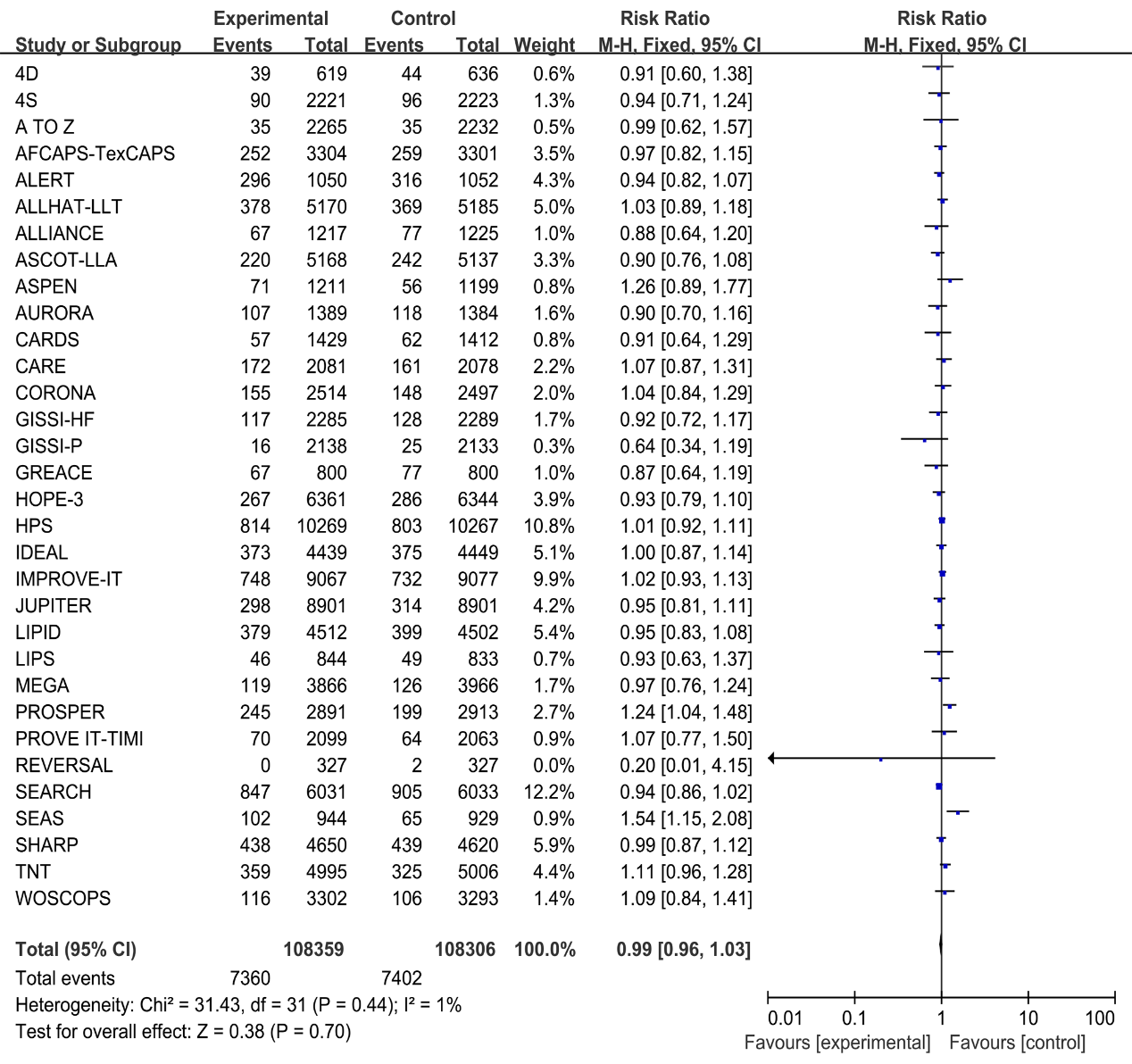


Figure S12. Forest plot of cancer in traditional meta-analysis.


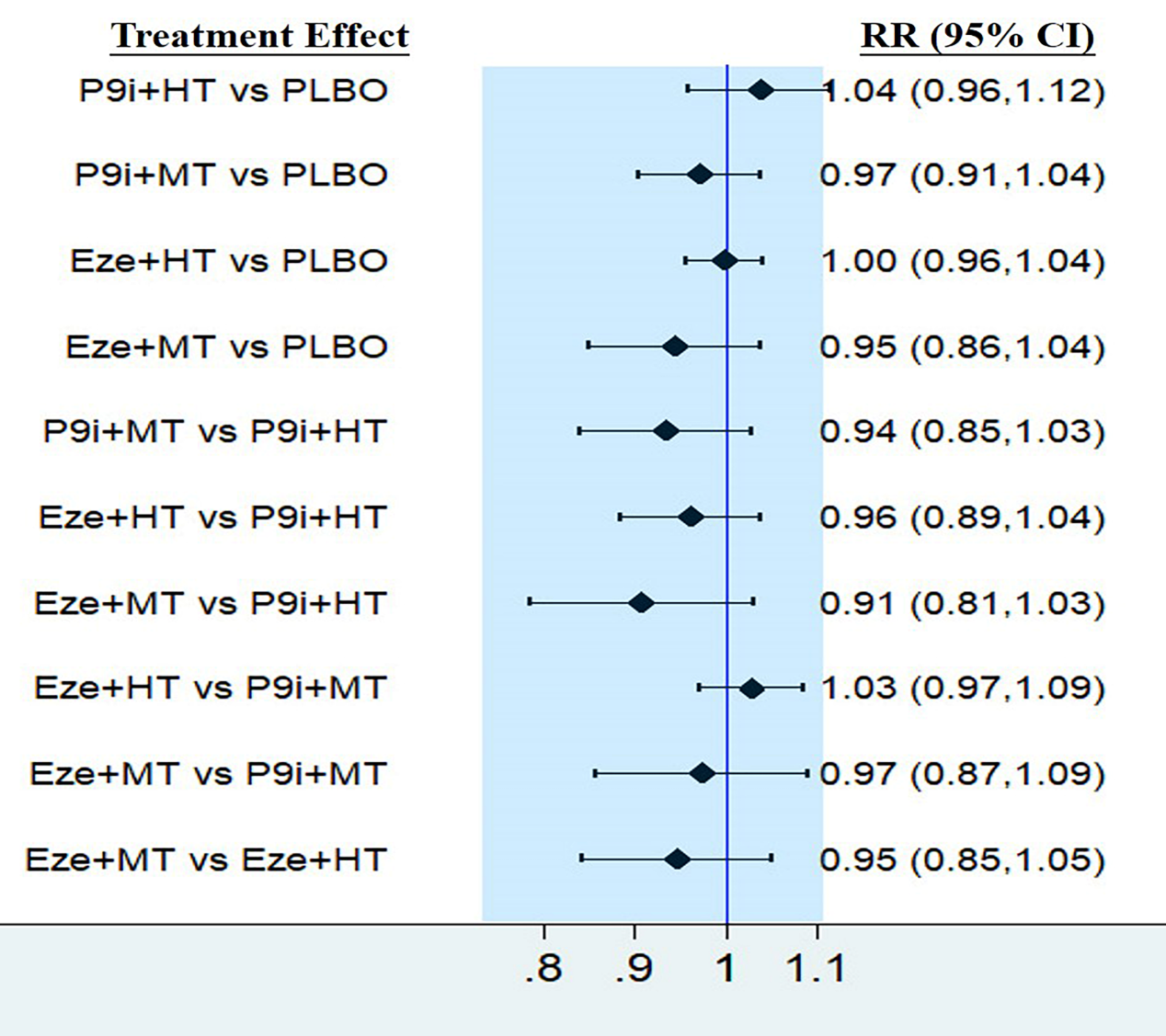


Figure S13. Forest plot of cancer in network meta-analysis.


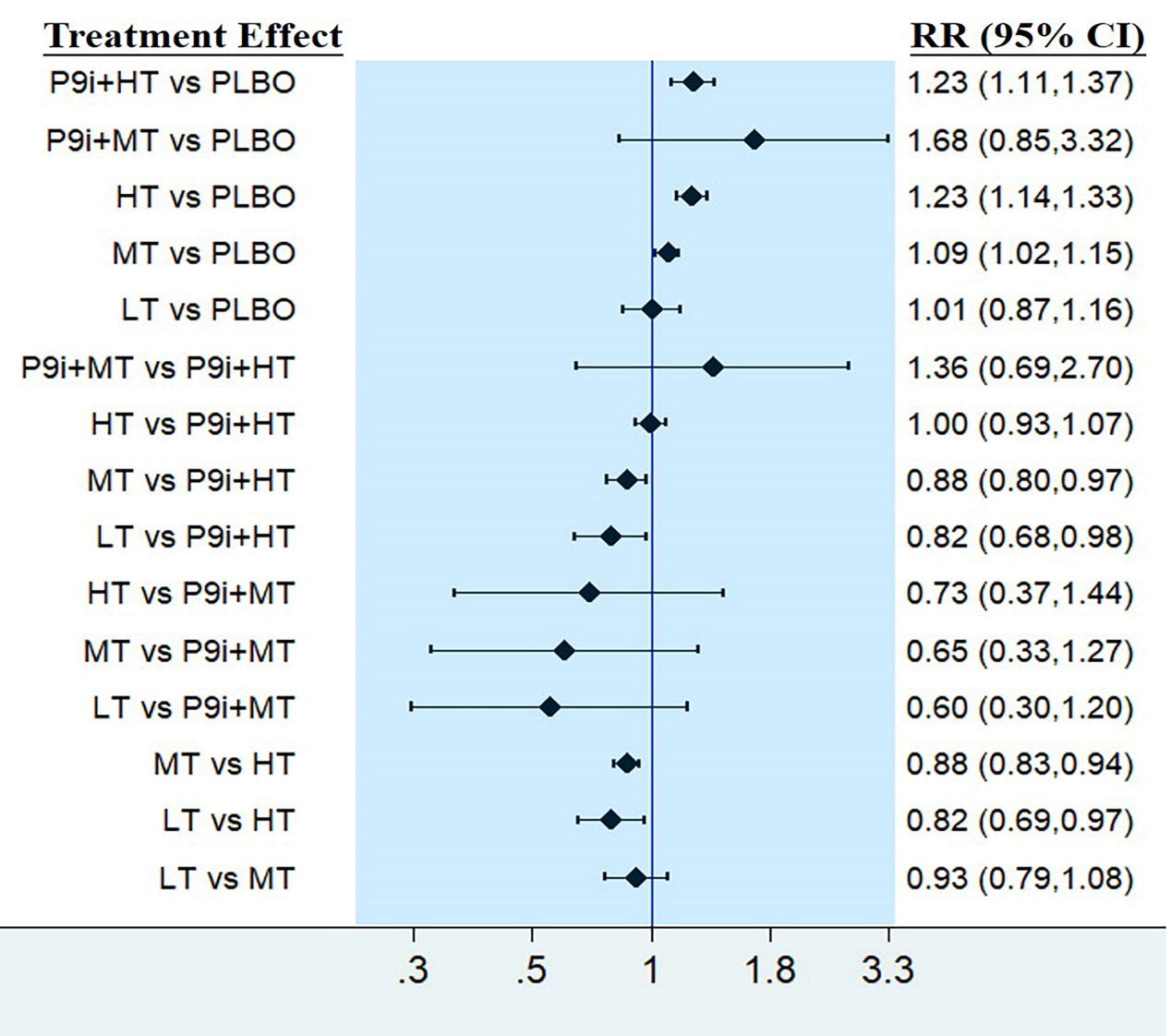


Figure S14. Forest plot of new-onset diabetes in network meta-analysis.

Table S1. Search strategies

| **PubMed** | | |
| --- | --- | --- |
|  | Searches | Results |
| #1 | ((statin[Title/Abstract])) OR (*statin[Title/Abstract]) | 24163 |
| #2 | ezetimibe[Title/Abstract] | 3345 |
| #3 | (((pcsk9[Title/Abstract])) OR (*pcsk9[Title/Abstract])) OR (pcsk9*[Title/Abstract]) | 3736 |
| #4 | #1 OR #2 OR #3 | 28611 |
| #5 | ((((((randomized controlled trial*[Title/Abstract])) OR (random*[Title/Abstract])) OR (controlled clinical trial*[Title/Abstract])) OR (trial*[Title/Abstract]) | 1832874 |
| #6 | #4 AND #5 | 8862 |
| **Embase** | | |
|  | Searches | Results |
| #1 | hydroxymethylglutaryl coenzyme a reductase inhibitor | 162853 |
| #2 | ezetimibe | 10535 |
| #3 | Proprotein convertase 9 | 4697 |
| #4 | #1 OR #2 OR #3 | 167514 |
| #5 | #4 AND ‘randomized controlled trial’ | 10783 |
| **Cochrane** | | |
|  | Searches | Results |
| #1 | MeSH descriptor:[Hydroxymethylglutaryl-CoA Reductase Inhibitor]explode all trees | 3545 |
| #2 | MeSH descriptor:[ezetimibe] explode all trees | 772 |
| #3 | (pcsk9 inhibitors):ti,ab,kw | 413 |
| #4 | (statin):ti,ab,kw | 9547 |
| #5 | (ezetimibe):ti,ab,kw | 1738 |
| #6 | #1 OR #2 OR #3 OR #4 OR #5 | 11606 |
| #7 | Trails | 11529 |
